# Geneformer-guided multiomics integration identifies Pbx1 as a network hub of hematopoietic stem cell aging

**DOI:** 10.1126/sciadv.aeb1346

**Published:** 2026-08-21

**Authors:** Hiroshi Kobayashi, Shintaro Watanuki, Yusuke Shiozawa, Motohiko Oshima, Shuhei Koide, Naoya Takayama, Takayuki Morikawa, Miho Haraguchi, Shinpei Tamaki, Takayoshi Asakura, Toshio Miyata, Atsushi Iwama, Seishi Ogawa, Keiyo Takubo

**Affiliations:** ^1^ Department of Cell Fate Biology and Stem Cell Medicine, Tohoku University Graduate School of Medicine, Sendai 980-8575, Japan.; ^2^ Department of Stem Cell Biology, National Institute of Global Health and Medicine, Japan Institute for Health Security (JIHS), Tokyo 162-8655, Japan.; ^3^ Laboratory of Molecular Analysis, Nippon Medical School, Tokyo 113-8602, Japan.; ^4^ Division of Stem Cell and Molecular Medicine, Center for Stem Cell Biology and Regenerative Medicine, The Institute of Medical Science, The University of Tokyo, Tokyo 108-8639, Japan.; ^5^ Department of Regenerative Medicine, Graduate School of Medicine, Chiba University, Chiba 260-8670, Japan.; ^6^ Kanagawa Institute of Industrial Science and Technology (KISTEC), Kawasaki 210-0821, Japan.; ^7^ Department of Molecular Medicine and Therapy, Tohoku University Graduate School of Medicine, Sendai 980-8575, Japan.; ^8^ Department of Pathology and Tumor Biology, Graduate School of Medicine, Kyoto University, Kyoto 606-8501, Japan.; ^9^ Institute for the Advanced Study of Human Biology (WPI-ASHBi), Kyoto University, Kyoto 606-8501, Japan.; ^10^ Department of Stem Cell Healthspan, Healthspan Research Center (HeSReC), Tohoku University, Sendai 980-8575, Japan.

## Abstract

Hematopoietic stem cells (HSCs) constitute an organized hematopoietic system that undergoes age-related alterations, including increased platelet production and decreased erythropoiesis. The fundamental mechanisms driving these shifts remain incompletely understood. We used single-cell RNA sequencing data to show that old HSCs contain two distinct transcriptional programs: one shared with megakaryocytes and the other reflecting the most primitive HSC state. Developmental time-series profiling further suggests that the acquisition of these programs begins early in life, with the primitive module rising prenatally and megakaryocytic priming emerging after birth. Using a fine-tuned Geneformer (transformer-based deep learning model) to capture higher-order differences between young and old HSCs, coupled with transcriptomic and epigenetic profiling, as well as transcription factor screens, we identified Pbx1 as a key regulator of these age-related transcriptional and differentiation changes. Specifically, Pbx1 suppresses erythroid differentiation by repressing Gata1 expression. These findings provide insight into HSC aging and may inform approaches to modulate age-associated HSC dysfunction.

## INTRODUCTION

Physiological aging of stem cells is a critical determinant of tissue homeostasis and maintenance of functional capacity throughout the life of an individual (*1*–*3*). While various factors have been implicated in stem cell aging, it is still unclear how the intrinsic transcriptional and epigenetic factor network is rewired during stem cell aging. Hematopoietic stem cells (HSCs) and their progeny form a well-defined stem cell system responsible for lifelong hematopoiesis through self-renewal and through generation of multipotent progenitors (MPPs) and committed progenitors (*4*). Similar to other types of stem cells, HSCs experience progressive alterations in their functional capabilities and phenotypic characteristics as they age. They exhibit a propensity for differentiating into platelets and myeloid cells (*5*–*7*), decreased differentiation into erythrocytes and lymphocytes (*8*), increased self-renewal, and expansion of their numbers, accompanied by an augmented susceptibility to hematological disorders (*5*, *9*–*11*). The induction of HSC aging is a multifaceted process influenced by the interplay between external environmental signals and internal cellular changes. Seminal single-cell lineage-tracing and barcoding studies have demonstrated that transcriptional priming toward megakaryocyte is already detectable in immunophenotypic HSCs of young mice (*12*, *13*). These findings imply that lineage bias is encoded early and raise the question of how this preexisting architecture is remodeled with age. Although epigenetic drift (*7*, *9*, *14*–*24*), replication stress (*21*), chronic inflammation (*16*, *25*, *26*), and altered cytokine microenvironment (*15*) have each been implicated in rewiring the HSC gene-regulatory network (GRN) during aging, how these external inputs converge on intrinsic transcriptional hubs remains obscure. Here, we ask whether a discrete set of “hub” factors can account for both the self-renewal advantage and lineage bias of old HSCs. Although the inhibition or restoration of environmental and epigenetic factors can partially rejuvenate the network (*25*–*29*), the observation that old HSCs do not rejuvenate when returned to younger hosts (*30*, *31*) suggests that alterations in this higher-order GRN are hardwired. Therefore, identifying hub factors that control this transcriptional network is critical to understanding stem cell aging and exploring approaches to rejuvenation.

Recently accumulating single-cell data have enabled construction of genome-scale GRNs (*32*). Gene network models employing deep neural networks offer robust tools for cell type classification and imputation of GRN, likely due to their flexibility in embedding higher-order architecture of GRNs in latent space (*33*). Among them, transformer/Bidirectional Encoder Representations from Transformers (BERT) architectures, originally developed for natural language processing, have shown high classification performance with different cell types (*34*–*40*). In this study, we used a BERT-based foundation model trained on an extensive dataset of cells and applied transfer learning with single-cell RNA sequencing (scRNA-seq) data from young and old hematopoietic stem and progenitor cells (HSPCs) to construct a model that embeds the GRN with high accuracy in classifying closely related HSPC cell types while accounting for age-related differences. By integrating this model with epigenomic analyses and extensive transcription factor (TF) screenings, we were able to show that the observed differentiation bias in old HSCs is due to Pre-B-cell leukemia homeobox 1 (Pbx1)-mediated activation of homeobox (HOX) regulatory elements. This activation is responsible for delayed expression of differentiation markers, such as CD48, following cytokine stimulation and results in reduced Gata1 induction, leading to reduced erythroid output. These findings indicate that age-related changes in HSCs are closely linked to stem cell identity and that activation of the core network triggers age-related alterations. Therefore, identifying and functionally interrogating hub factors within this central aging/stem-cell network may provide a framework for rationally modulating age-associated HSC phenotypes.

## RESULTS

### Old HSCs coexist with transcriptional features of both the most primitive HSCs and megakaryocytic progenitors

**Fig. 1.**
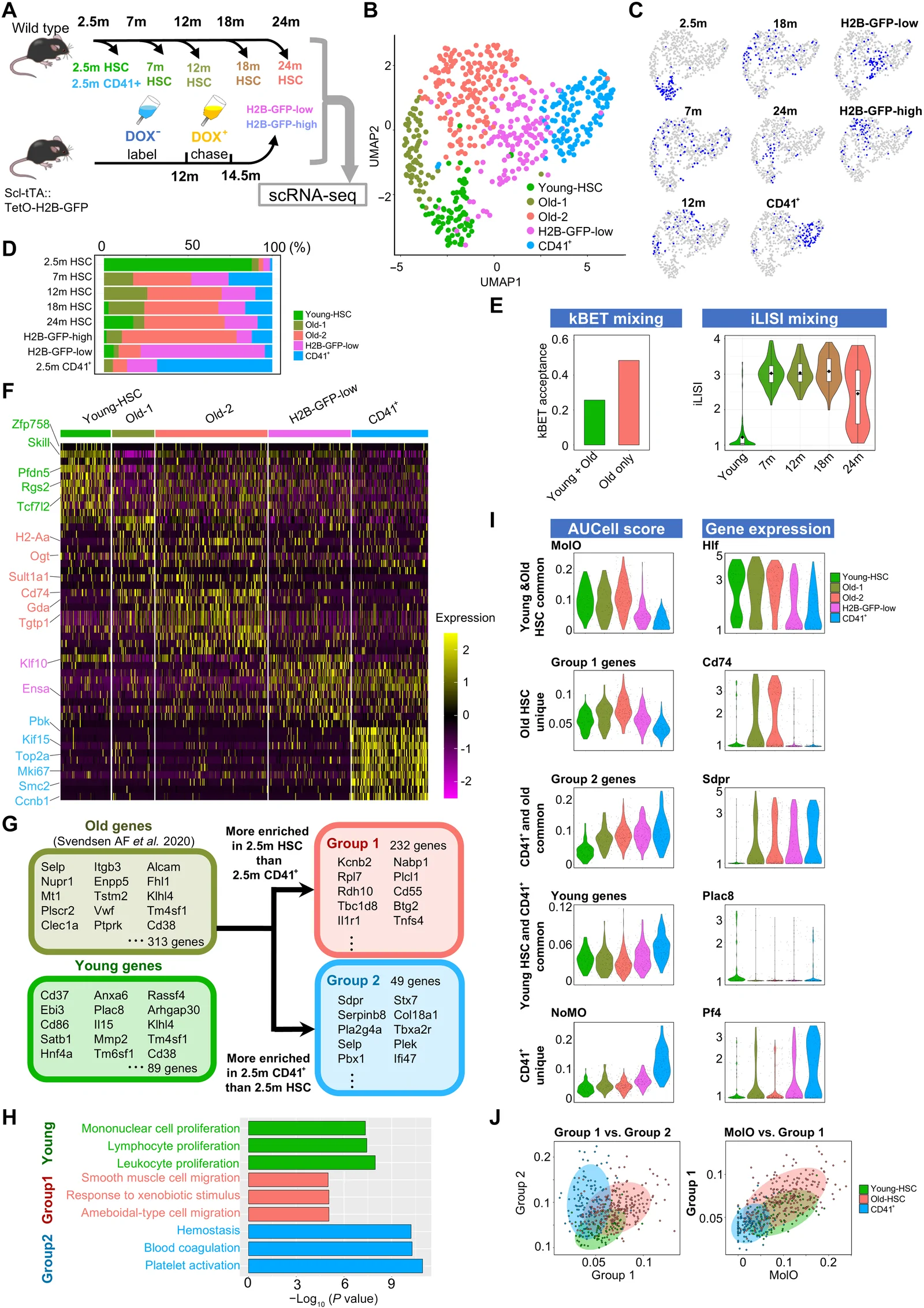
Old HSCs coexist with transcriptional features of both the most primitive HSCs and megakaryocytic progenitors. (**A**) Schematic showing single cell RNA-seq analysis. Data are from one (except for H2B-GFP low and CD41^+^ SLAM-LSK cells) or two (H2B-GFP low and CD41^+^ SLAM-LSK cells) samples. (**B**) UMAP of scRNA-seq data. Data are clustered and labeled by Seurat. Each cluster was named after the main population that composes the cluster. (**C**) Cells of each original group projected on UMAP. (**D**) Distribution of clusters within the original group. (**E**) kBET mixing (left) and iLISI mixing (right) analyses. (**F**) Heatmap showing genes characteristic of each cell type. (**G**) Classification of age-associated genes. Old HSC-specific genes were categorized into Group 1 (enriched in primitive HSCs versus CD41^+^ cells) and Group 2 (shared with megakaryocytic CD41^+^ cells). Genes consistently up-regulated in either young or old HSCs in 3 or more of the 12 datasets [reanalyzed by Svendsen AF *et al.* (*41*)] were obtained. Genes not detected by scRNA-seq were removed. (**H**) Gene Ontology analysis of young HSC genes, group1 old HSC genes, and Group2 old HSC genes. (**I**) AUCell scores or expression levels characteristic of the indicated five groups are shown. *P* values are provided in table S1. (**J**) Dual-signature analysis at the single-cell level. Scatter plots demonstrate the simultaneous enrichment of Group 1/MolO signatures and Group 2 signatures within individual old HSCs. Approximately 80% of cells are circled for each group.

Aging induces consistent changes in gene expression of HSCs (*41*). However, it remains unclear whether these deregulated aging genes can be further subdivided. Therefore, we sought to classify age-related genes based on timing of gene expression changes, and their relationship to the cell cycle. To address this, we performed scRNA-seq on (i) HSCs at different ages, (ii) CD41^+^CD150^+^CD48^−^lineage^−^c-Kit^+^Sca1^+^ (CD41^+^ signaling lymphocytic activation molecule (SLAM)-LSK) cells from young mice, and (iii) populations with high and low expression of the cell division history marker H2B-GFP (Fig. 1A and fig. S1A). As a result, cells were classified into five clusters: Young-HSC, Old-1, Old-2, H2B-GFP-low, and CD41^+^ (Fig. 1B). Cluster names were assigned on the basis of the dominant source population within each cluster. Despite their marker similarity, young cells with a marker phenotype of CD41^+^ SLAM-LSK cells formed a distinct cluster from the CD41^−^ HSC fraction from young mice (young HSCs) (Fig. 1, B and C). The analysis of scRNA-seq data in public databases (dataset 2 and dataset 3) also confirmed that a population characterized by high expression of *Itga2b*, a gene encoding CD41, as well as cell cycle–related S and G_2_-M genes in phenotypic HSCs, together with S- and G_2_-M–phase cell cycle genes, formed a cluster containing both young and old cells (fig. S1, B to D). On the other hand, HSCs from 7 to 24 months of age and H2B–green fluorescent protein (GFP) label-retaining HSCs were divided into two clusters, designated Old-1 and Old-2, with similar distribution among aged population (Fig. 1D). H2B-GFP low HSCs were located between old HSCs and CD41^+^ SLAM-LSK cells with respect to cell cycle gene expression and the immature HSC-related MolO signature (fig. S1D), consistent with the reduced repopulation capacity of nonlabel-retaining HSCs (*42*–*44*). The gene expression profile of 7-month-old HSCs and H2B-GFP-high HSCs was almost indistinguishable from that of 24-month-old HSCs when comparing cell cycle genes and MolO signature (Fig. 1D and fig. S1E; *P* values are summarized in table S1, including subsequent datasets). This observation was further supported by a higher mixing rate in a *k*-nearest neighbor batch effect test (kBET) analysis (*45*) and a higher local inverse Simpson’s index (LISI) (*46*) among 7- to 24-month-old HSCs than between young and 7- to 24-month-old HSCs (Fig. 1E). Each cluster displayed a distinct gene expression profile (Fig. 1F).

To characterize the gene expression programs defining each cluster, we classified 313 genes reproducibly up-regulated across multiple transcriptomic datasets (*41*) into two groups based on their relative expression in young HSCs and CD41^+^ SLAM-LSK cells: genes with comparable or higher expression in young HSCs (Group 1 old HSC genes; Group 1 genes) and genes with higher expression in young CD41^+^ SLAM-LSK cells (Group 2 old HSC genes; Group 2 genes) (Fig. 1G). Gene Ontology (GO) analysis revealed that Group 2 genes are enriched for platelet and coagulation-related terms (Fig. 1H), whereas Group 1 genes are largely devoid of platelet-associated genes and were enriched for leukocyte proliferation, glutathione synthesis, immune receptors, and phospholipid transporters (Fig. 1H and fig. S1F).

When we mapped the resulting young genes, Group 1 genes, and Group 2 genes together with the MolO signature that defines HSC function and the NoMO signature predicting non-repopulating cells (*47*), the following five programs were identified: a program common to young and old HSC populations (MolO), a program specific to old HSC population (Group 1 genes), a program common to young CD41^+^ SLAM-LSK cells and old HSC populations (Group 2 genes), a program common to young HSCs and CD41^+^ SLAM-LSK cells (Young genes), and a program specific to the CD41^+^ SLAM-LSK cells (NoMO) (Fig. 1I and fig. S1G; see table S2 for gene lists). Old-1 cells displayed lower expression of Group 1 genes than Old-2 cells. However, this heterogeneity among old cells was not observed in the reanalyzed datasets, dataset 2 and dataset 3 (Fig. 1I and fig. S1C). Both old and young HSCs showed the high level of Hlf expression, characteristic of the most primitive HSCs (*48*), whereas young CD41^+^ SLAM-LSK cells and H2B-GFP low fractions exhibited a high level of the NoMO signature characteristic of nonrepopulating cells (*47*) and also expressed high levels of the megakaryocyte/platelet differentiation marker *Pf4* (Fig. 1F and fig. S1D) (*49*), in line with their actively cycling status. When Group 1 genes and Group 2 genes or the MolO signature were plotted together, old HSCs showed simultaneous enrichment of Group 1 genes, Group 2 genes, and the MolO signature at the single-cell level, whereas CD41^+^ cells were enriched only for the Group 2 signature and young HSCs only for the MolO signature (Fig. 1J and fig. S1H). These features indicate that old HSCs simultaneously activate two distinct programs associated with primitive HSCs and megakaryocytic progenitors, partly consistent with a functional bias toward platelet differentiation in old HSCs (*8*).

Next, we analyzed transcriptomes of HSCs in early development (*50*) to determine when each of these programs is triggered. The expression of Group 1 genes began to increase as early as prenatal embryonic day 18.5 (E18.5). In contrast, the expression of Group 2 genes began to increase at 4 to 8 weeks of age, and the expression of young HSC genes decreased during the same period. These observations suggest that acquisition of old HSC-associated gene signatures is not a phenomenon specific to the elderly, but rather a continuous process that begins early in life, including during pregnancy (fig. S1I).

In summary, whereas HSCs from different old cohorts (7 to 24 months) displayed similar transcriptional profiles, in contrast to the marked differences observed between young and old HSCs, many individual old HSCs show concurrent enrichment of two distinct programs: one associated with undifferentiated HSCs (Group 1 genes and the MolO signature) and the other associated with the megakaryocyte/platelet lineage (Group 2 genes).

### Impaired differentiation-linked chromatin remodeling and persistent accessibility of age-associated regulatory regions in old HSCs

**Fig. 2.**
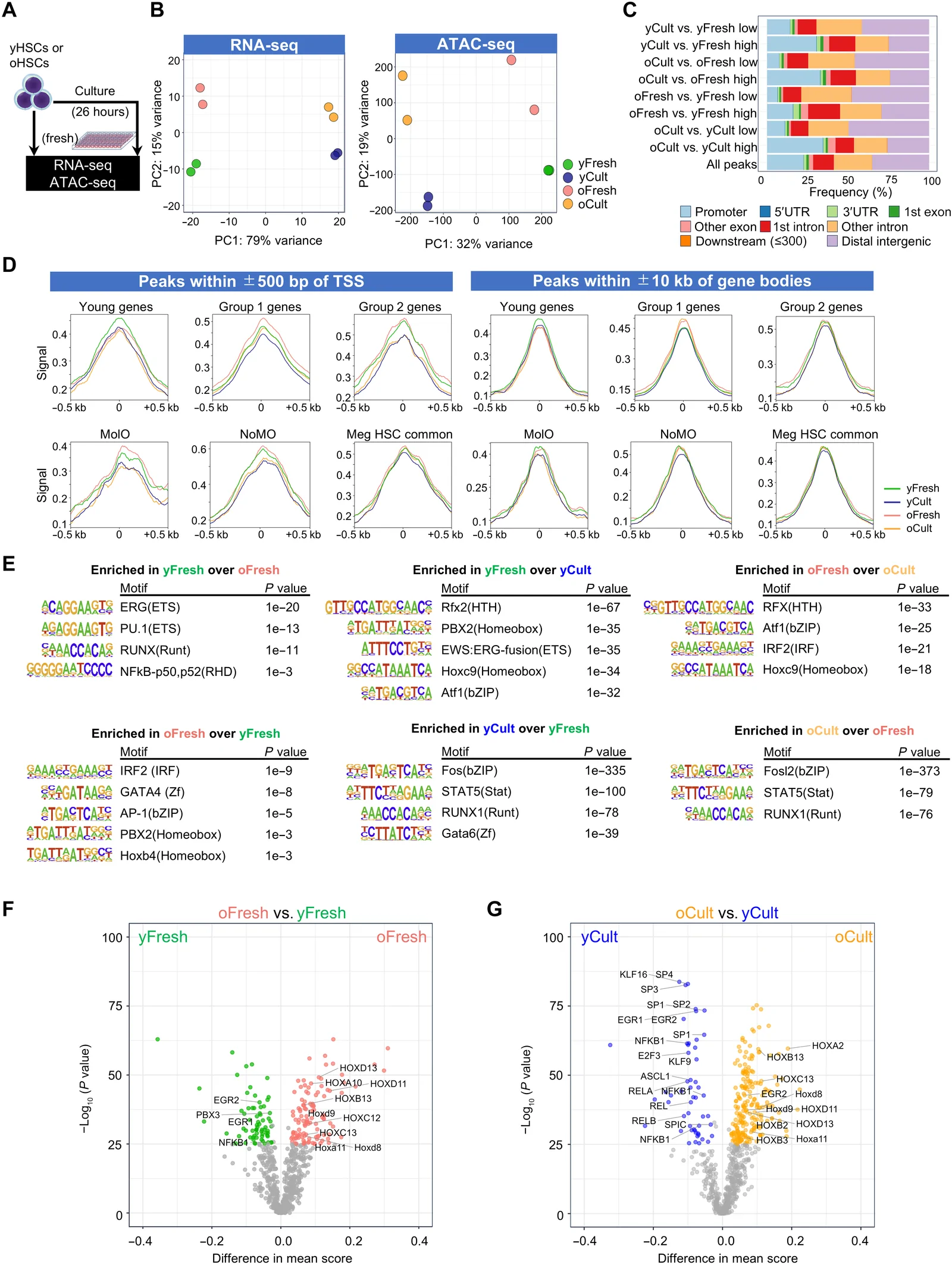
Old HSCs are defective in activating differentiation programs after cytokine stimulation. (**A**) Experimental design. Twelve young mice (14 weeks old) and two old (25 months and 28 months old) mice were pooled in each replicate (*n* = 2 biological replicates per group). (**B**) PCA of RNA-seq and ATAC-seq using normalized read counts. (**C**) The percentage of annotated genomic regions on which each DAR locates. (**D**) Line plots of ATAC-seq signal profiles centered on TSSs or peaks within ±10 kb of the nearest genes, including gene bodies of young or old HSC-associated genes and genes comprising the MolO, NoMO, and Meg HSC common gene sets (table S2) with 1-kb windows. (**E**) HOMER-predicted TF binding motifs enrichment comparing indicated cell types. (**F**) TOBIAS-predicted enriched TF footprints comparing young fresh and old fresh HSCs. Motifs with adjusted *P* value (*P*adj) < 1 × 10^−25^ and an absolute difference in mean score > 0.2 are considered as significant. (**G**) TOBIAS-predicted enriched TF footprints comparing young cultured and old cultured HSCs. 3′UTR, 3′ untranslated region; 5′UTR, 5′ untranslated region.

Next, we investigated the relationship between transcriptional changes in old HSCs and their epigenetic state. Characteristics such as reduced engraftment efficiency and differentiation skewing become most apparent in old HSCs when subjected to proliferative stimuli after transplantation (*20*, *51*, *52*). To investigate the earliest changes in the program of old HSCs after proliferative stimulation, we performed RNA-seq and assayed for transposase-accessible chromatin sequencing (ATAC-seq) (*53*) simultaneously after 26 hours of cytokine stimulation (Fig. 2A and see table S3 for alignment metrics), a time point at which approximately half of young HSCs have completed their first division (*21*). Freshly isolated HSCs from young or old mice (yFresh or oFresh) and young or old HSCs after cytokine stimulation (yCult or oCult) with two essential cytokines for HSCs, stem cell factor (SCF), and thrombopoietin (TPO), each group formed distinct clusters in the principal components analysis (PCA) plot in either RNA-seq or ATAC-seq analysis (Fig. 2B). In this analysis, PC1 primarily reflects the presence or absence of cytokine stimulation, whereas PC2 captures age-associated differences (Fig. 2B). A notable proportion of aging-associated differentially expressed genes (DEGs) (fig. S2, A to C) and differentially accessible regions (DARs) (fig. S2, D and E) are maintained after cytokine stimulation. Similarly, 40 to 60% of culture-associated DEGs and DARs overlap in yFresh and oFresh HSCs. Relative to their young counterparts, oFresh HSCs exhibited a modest increase in promoter-associated DARs, although these regions remained predominantly located in distal intergenic areas. In contrast, oCult DARs showed a more pronounced enrichment in promoter regions when compared with yCult HSCs (Fig. 2C).

To evaluate the relationship between age-associated gene programs and epigenetic status, we analyzed aggregated ATAC-seq signal at promoters and at distal peaks (defined as peaks within the gene body and ±10 kb, including enhancers) whose nearest genes belonged to the following gene sets: Young genes, Group 1 genes, Group 2 genes, MolO, NoMO, and a set of genes shared between megakaryocytes and HSCs but not expressed in granulocyte/macrophage progenitors, common lymphoid progenitors, or erythroid cells (Meg HSC common genes; table S2 and Fig. 2D). Young genes showed the strongest enrichment in yFresh at both promoters and distal peaks. In contrast, Group 1 genes displayed higher signal at transcription start site (TSS) regions in oFresh, which decreased in oCult, whereas distal peaks exhibited high signal in both oFresh and oCult. Unlike Group 1 genes, Group 2 genes and Megakaryocyte HSC common genes showed reduced ATAC signal upon culture without an age-dependent difference. The MolO and NoMO signatures decreased with culture at both TSS regions and distal peaks.

To further understand the identified DARs, we predicted TF-binding motifs characteristic of young or old HSCs using HOMER (*54*). While yFresh HSCs are enriched for differentiation-associated Pu.1 (*55*) or Runx (*56*) motifs (Fig. 2E), oFresh HSCs are enriched in interferon regulatory factor (IRF), activator protein-1 (AP-1), and Homeobox motifs (Fig. 2E). Among these motifs, the Homeobox motif was specifically down-regulated in the cultured cells either in young or old samples (Fig. 2E). On the other hand, footprint analysis using TF Occupancy prediction By Investigation of ATAC-seq Signal (TOBIAS) revealed that both oFresh and oCult groups were overrepresented for HOX motifs (Fig. 2F), whereas yCult group was enriched for Sp1 and nuclear factor κB (NF-κB) sites, indicating active transcription (*57*) and differentiation program (*58*) (Fig. 2G). ChromVAR analysis (*59*) also confirmed the enrichment of HOX motifs in either oFresh or oCult compared to the young group and underrepresented HOX motifs after cytokine stimulation, whereas yCult exhibited enrichment of Pu.1 (Sfpi1 and Spic) motif relative to oCult (fig. S2F).

These findings, when integrated with the single-cell transcriptomic profiles in Fig. 1, describe a state of relative epigenetic rigidity in old HSCs. Unlike young HSCs, old HSCs exhibit lower accessibility at differentiation-linked loci, such as Pu.1 or NF-κB sites, which is evident in the fresh state and remains largely unchanged after cytokine stimulation. Simultaneously, old HSCs maintain high accessibility at genomic regions associated with Group 1 genes and HOX motifs regardless of the proliferative stimulus. This observation of a stable, age-associated epigenetic landscape suggests that the old HSC phenotype is governed by a coordinated regulatory framework that persists during the early stages of activation.

### Fine-tuning of a pretrained Geneformer enables identification of key transcriptional networks coordinating dual aging signatures

Changes in the transcriptome and epigenome are reflected as changes in higher-order GRN. A critical challenge arising from our findings in Figs. 1 and 2 is identifying the core regulatory logic that coordinates the aging-associated program linked to more primitive phenotypes (Group 1) and the megakaryocyte-biased program (Group 2) within individual old HSCs.

Because traditional gene-centric analyses often struggle to capture the complex, contextual dependencies of such a dual program, we applied Geneformer, a transformer-based deep learning framework (*34*, *60*), to identify the central regulatory hubs of this network. In this model, each gene is represented as a “token,” similar to a word in a sentence, and the model learns how genes tend to appear together across thousands of single-cell transcriptomes. Just as a language model captures the contextual relationships between words, Geneformer learns the context-dependent coactivation of genes, providing a quantitative map of how genes communicate with one another. The resulting attention weights can be interpreted as the strength of these regulatory relationships, allowing us to infer a GRN that reflects the transcriptional logic of young and old HSCs (an overview of the analytical workflow is summarized in fig. S3).

**Fig. 3.**
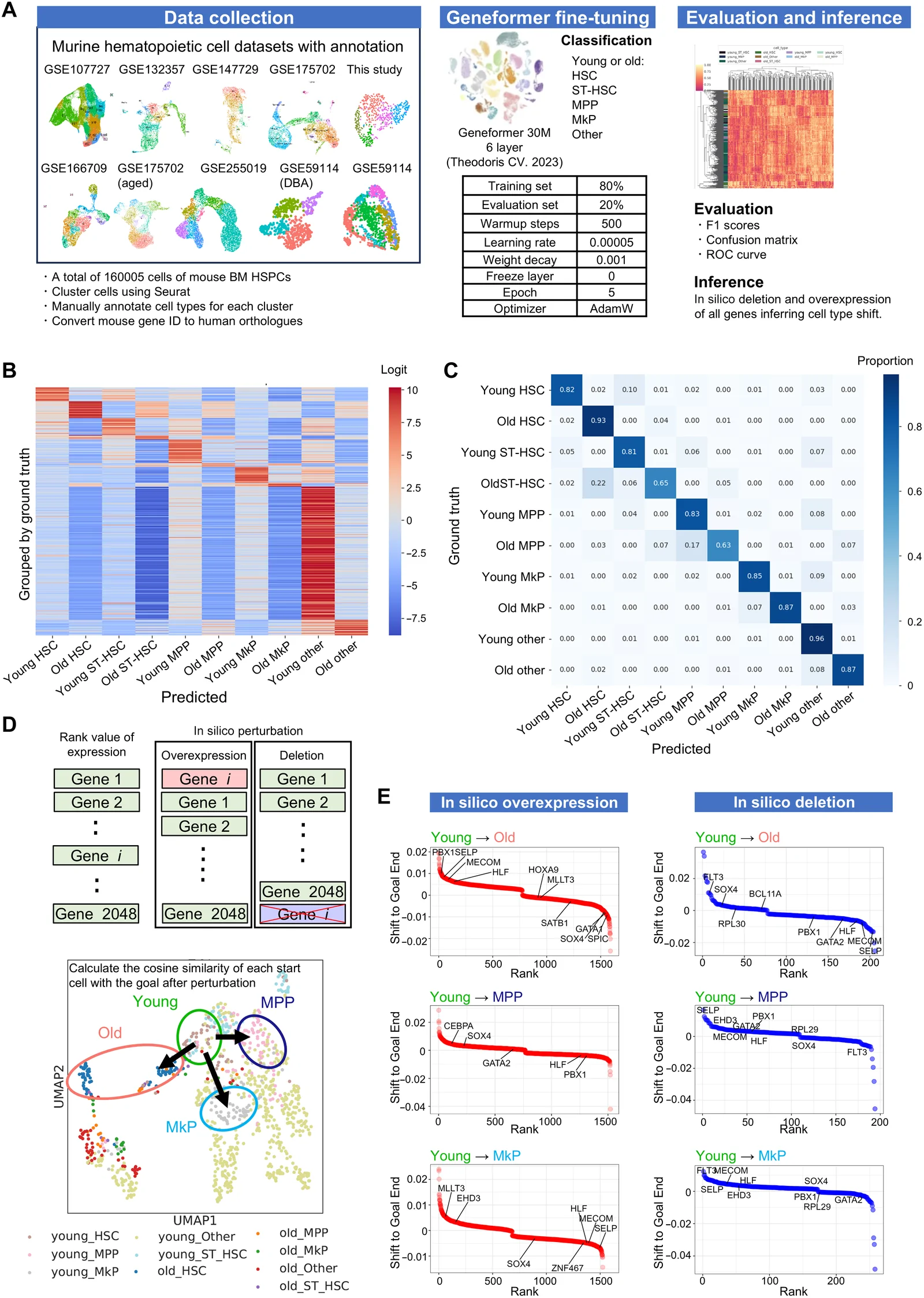
Fine-tuning of a pretrained Geneformer enables identification of key transcriptional networks in old HSCs. (**A**) Fine-tuning of Geneformer and deep learning-based discovery. Dataset for fine-tuning, training parameters, and evaluation and inference. (**B**) Heatmap representation of the logit scores for class predictions by the indicated models grouped, organized according to the ground truth. (**C**) Confusion matrix for classification of each cell type using the fine-tuned model. (**D**) Illustration of in silico perturbation (top) and UMAP representation of the final layer of embedded data (bottom). Shift from starting cells (young HSCs, Young) to each goal end (old HSCs, Old; young MPP, MPP; young MkP, MkP) were calculated, and the significant genes were ordered according to the impact. (**E**) Estimated transcriptional deviation after in silico perturbation of overexpression or deletion. Shown are significant genes from young HSC to old HSC (top) young HSC to MPP (middle) and young HSC to MkP (bottom).

We integrated 10 datasets of either young or old HSPC scRNA-seq data including the one described in Fig. 1 and fig. S1 and fine-tuned Geneformer using three methods (Fig. 3A and fig. S4, A and B), with one model fine-tuned in a stepwise manner (six-layer two-step model) and the other two in a single-step fine-tuning (six-layer one-step and 12-layer one-step models, respectively). Although the two-step model showed high performance in classifying immature bone marrow cells (fig. S4C), *F*_1_ scores, the harmonic mean of precision and recall, for discriminating young and old HSPCs were inferior to one-step models, presumably due to the lower number of cells assigned to young HSCs in the second fine-tuning (fig. S4D), whereas AUCell scores were comparable among the three methods (fig. S4E). Considering the classification performance and computational load, we chose the six-layer one-step model as the model for downstream analysis. Although a portion of either young or old HSCs were misclassified as short-term HSCs (ST-HSC) or MPPs in each model (Fig. 3B and fig. S4F), the accuracy of old HSC classification reached 0.93 and 0.82 for young HSC (Fig. 3C), indicating that subtle transcriptional differences in the most primitive HSPC fractions (HSC, ST-HSC, MPP, and MkP) and aging status can be appropriately embedded in the model.

Using the model, we inferred regulatory genes that affect aging and differentiation status with in silico perturbation, as described in the original article (*34*): In overexpression, the gene of interest is ranked at the top of the gene list, whereas in deletion, the gene of interest is ranked at the bottom of the list and the deviation from the starting cell type to the target cell type is calculated (Fig. 3D). Genes such as Pbx1, Mecom, Selp, and Hlf are thought to shift the phenotype of young HSCs to old upon in silico overexpression, whereas Spic, Sox4, and Gata1 overexpression resulted in a shift to young status. Conversely, in silico deletion of most, if not all, of these genes resulted in the opposite phenotype (Fig. 3E). Notably, Selp overexpression reportedly induced an old HSC-like phenotype (*41*), whereas Satb1 expression ameliorated the old phenotype in HSCs (*61*), suggesting that the model faithfully recapitulates the experimental results. The model also predicted that overexpression of Cebpa or Gata2 induced differentiation toward MPP, and HSC genes such as Hlf or Mecom expression shifted the MkP phenotype to HSCs (Fig. 3E). The other two models showed similar results, although the 12-layer one-step model showed a slightly different rank order with respect to HSC TFs (fig. S4H).

### Identification of Pbx1 as a key TF that confers age-related transcriptional features upon HSCs

To drill down factors that regulate the aging phenotype, we attempted to screen for TFs that recapitulate aspects of HSC aging. First, we searched for surface markers that could readily distinguish old from young HSCs after cytokine stimulation (fig. S5A). Among the surface markers tested, we focused on CD48, one of the earliest markers of HSC differentiation into MPP (*62*). While most of the young HSC-derived cells began to express CD48 on day 2 of culture, old HSC-derived cells showed delayed expression, with the difference becoming even greater on day 6 (fig. S5, A to C). The delayed expression of CD48 was also confirmed in the in vivo proliferative condition induced by transplantation (fig. S5D). The frequency of CD150^+^ CD48^−^ LSK fraction in bone marrow and spleen was significantly higher in recipients transplanted with old HSCs (fig. S5, E and F). ATAC-seq results showed that there is a genomic region upstream of the CD48 gene containing a signal transducers and activators of transcription (STAT)–binding motif that is opened upon cytokine stimulation and is more open in young HSCs (fig. S5G).

**Fig. 4.**
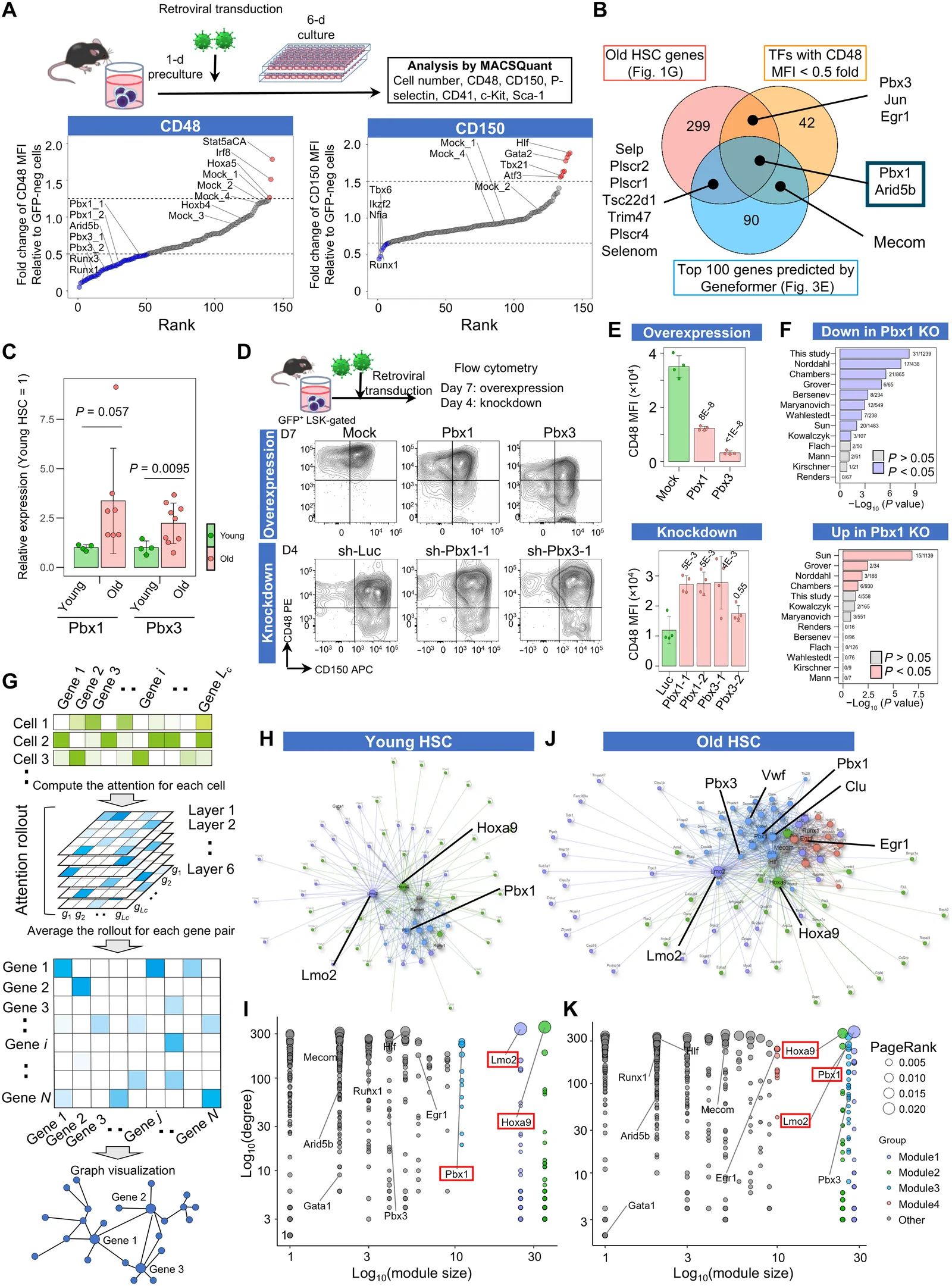
Identification of Pbx1 as a key TF that confers age-related transcriptional features upon HSCs. (**A**) Screening of TFs using retroviral expression. Each dot represents a fold change of indicated markers in GFP^+^ cells compared to nontransduced GFP-cells in the same culture well. Results are derived from a combination of three experiments. (**B**) Shared genes between old HSC genes (Fig. 1G), TFs that down-regulated CD48 expression, and top 100 genes predicted by fine-tuned Geneformer (Fig. 3E). (**C**) qPCR analysis of Pbx1 or Pbx3 comparing young and old HSCs (means ± SD, *n* = 4 to 9 biological replicates for each group from three experiments). Unpaired two-tailed *t* test. (**D**) Representative FACS plots of the LSK fraction following retroviral overexpression (top) or knockdown (bottom) of Pbx1 or Pbx3. Mock, mock-IRES-GFP. Luc, luciferase. (**E**) Quantification of (**D**) (means ± SD, *n* = 4 wells per group from one experiment). *P* values are shown above the bars; not significant (n.s.); Tukey’s multiple comparisons test. (**F**) Hypergeometric test on overlapping genes between old HSC specific genes in indicated transcriptomic dataset, and genes down-regulated (top) or up-regulated (bottom) in Pbx1-deficient HSCs [data adapted from (*65*)]. Transcriptomic data comparing young and old HSCs were sourced from AS web tool site (https://agingsignature.webhosting.rug.nl/). Data of this study correspond to scRNA-seq data presented in Fig. 1. (**G**) Schematic illustration of attention rollout computation and network visualization. (**H**) Network structure of young HSCs. Genes belonging to large modules (colored) are shown. (**I**) Network metrics of young HSC, including Degree, PageRank, and size of module to which each gene belongs are shown. Modules with the number of genes >9 are colored. (**J**) Network structure of old HSCs. (**K**) Network metrics of old HSC. KO, knockout.

Using changes in CD48 expression as a surrogate for progression of differentiation, we searched for genes involved in delaying cytokine-stimulated differentiation in old HSCs. We screened for candidate genes by introducing a custom retroviral library of 143 hematopoiesis-related TFs and regulators (table S4) into young HSCs. Comparison of HSC-derived cells transduced with candidate factors with nontransduced cells by flow cytometry identified genes including a constitutively active form of STAT5 (Stat5aCA) (*63*), Irf8, and Hoxa5 as factors that up-regulated CD48 expression more than twofold and genes including Runx1, Runx3, Pbx1, and Pbx3 as factors that down-regulated CD48 expression less than 0.5-fold (Fig. 4A). Genes including Hlf up-regulated CD150 consistent with its specific function in HSC maintenance (*48*, *64*), whereas Runx1 and Runx3 suppressed CD150 expression (Fig. 4A), indicating their function in promoting HSC differentiation.

Combining this TF screening, the inference from the Geneformer data (Fig. 3E) and genes highly expressed in old HSCs (Fig. 1G), we identified Pbx1 and Arid5b as TFs that may contribute to the aging phenotype of HSCs (Fig. 4B). Pbx1 was up-regulated in old HSCs in both our dataset and those in public databases (fig. S5H) and was validated by quantitative polymerase chain reaction (qPCR) of independent old HSC samples (Fig. 4C), whereas the expression of Arid5b was low in public datasets (fig. S5H). Considering that Pbx1 functions as a heterodimer with Homeobox binding proteins that is enriched in cytokine-stimulated old HSCs (Fig. 2G and fig. S2F), we further investigated the function of Pbx1. Notably, Pbx3, a paralog of Pbx1 and a Group 2 gene, was expressed at lower levels than Pbx1 (fig. S5H).

To validate the screening results, we induced either the overexpression or the knockdown of Pbx1 and Pbx3 in young HSCs. The expression levels at protein (for Pbx1; fig. S5I) and transcript (table S5) confirmed the up- or down-regulation of each gene. Pbx1 overexpression again reduced CD48 expression, recapitulating the expression pattern observed in old HSCs. Conversely, the knockdown of Pbx1 or Pbx3 with short hairpin RNA (shRNA)–based retroviral vectors in old HSCs up-regulated CD48 expression (Fig. 4, D and E). Furthermore, we found that a significant proportion of genes that were down-regulated after Pbx1 deletion in HSCs (*65*) were also up-regulated in old HSCs in 9 of the 12 datasets analyzed, both ours and those publicly available (Fig. 4F).

While our experimental screening and functional validation established Pbx1 as a potent regulator of the old phenotype, the dual signature of old HSCs (primitive HSC and platelet programs) suggests that Pbx1 may function as a central hub within a larger GRN. Although our fine-tuned Geneformer model successfully predicted Pbx1 as a key factor, transformer-based architectures are inherently “black boxes,” making it difficult to directly observe the high-dimensional relationships they learn. To move beyond individual gene assessments and interpret the global regulatory logic learned by the model, we sought to extract the underlying GRN structure. By quantifying the strength of gene-gene associations through the model’s internal weights, we can transform these deep learning representations into a biologically interpretable map of aging-specific interactions.

Since the transformer architecture is characterized by its attention mechanism, which assigns weights to token-token relationships (*66*), attention weights provide a model-derived measure of context-dependent gene-gene association. These associations do not by themselves establish direct physical or regulatory interactions; we therefore use them to prioritize candidate hub genes for experimental validation. Accordingly, we used the attention rollout method (*67*) to extract gene-gene association weights and visualized subgraphs comprising young HSC genes, old HSC genes, and hematopoiesis-associated TFs (Fig. 4, G to K, and table S6). Following subgraph extraction, we calculated module size and assessed centrality using node degree and PageRank metrics (*68*, *69*) to identify hub genes in the aging-associated network. The analysis of both young and old HSCs demonstrated that the Hoxa9 and Lmo2 modules are preserved throughout aging, whereas the Pbx1 module, which includes age-associated genes such as Clu and Vwf, exhibits specific expansion in old HSCs (Fig. 4, H and J). Both centrality measures yielded consistent results, and HSC-specific TFs such as Hlf and Mecom generally have high centrality, albeit forming smaller modules compared with Hoxa9, Lmo2, and Pbx1 (Fig. 4, I and K). Together, these analyses support Pbx1 as a prominent hub within the aging-associated network in old HSCs.

To further understand the function of Pbx1 within the transcriptional network of old HSCs, we also performed weighted correlation network analysis (WGCNA) (*70*) with the same gene set. This network consisted of three clusters—core HSC TFs, megakaryocyte (Meg) genes, and old HSC genes—based on the genes included (fig. S5J). The Core HSC TF network contained HSC-specific genes such as Hlf and Mecom, whereas the Meg gene network contained exclusively megakaryocytic genes such as Gata1 and Runx1. Notably, Pbx1 was positioned at the interface of these three clusters and established connections among them (fig. S5K). This suggests that Pbx1 is essential in the gene expression network in old HSCs and can explain the dual nature of the old HSC phenotype, which shows platelet bias and up-regulation of HSC-specific genes.

### Pbx1 activates a core transcriptional network shared by aging and stemness

**Fig. 5.**
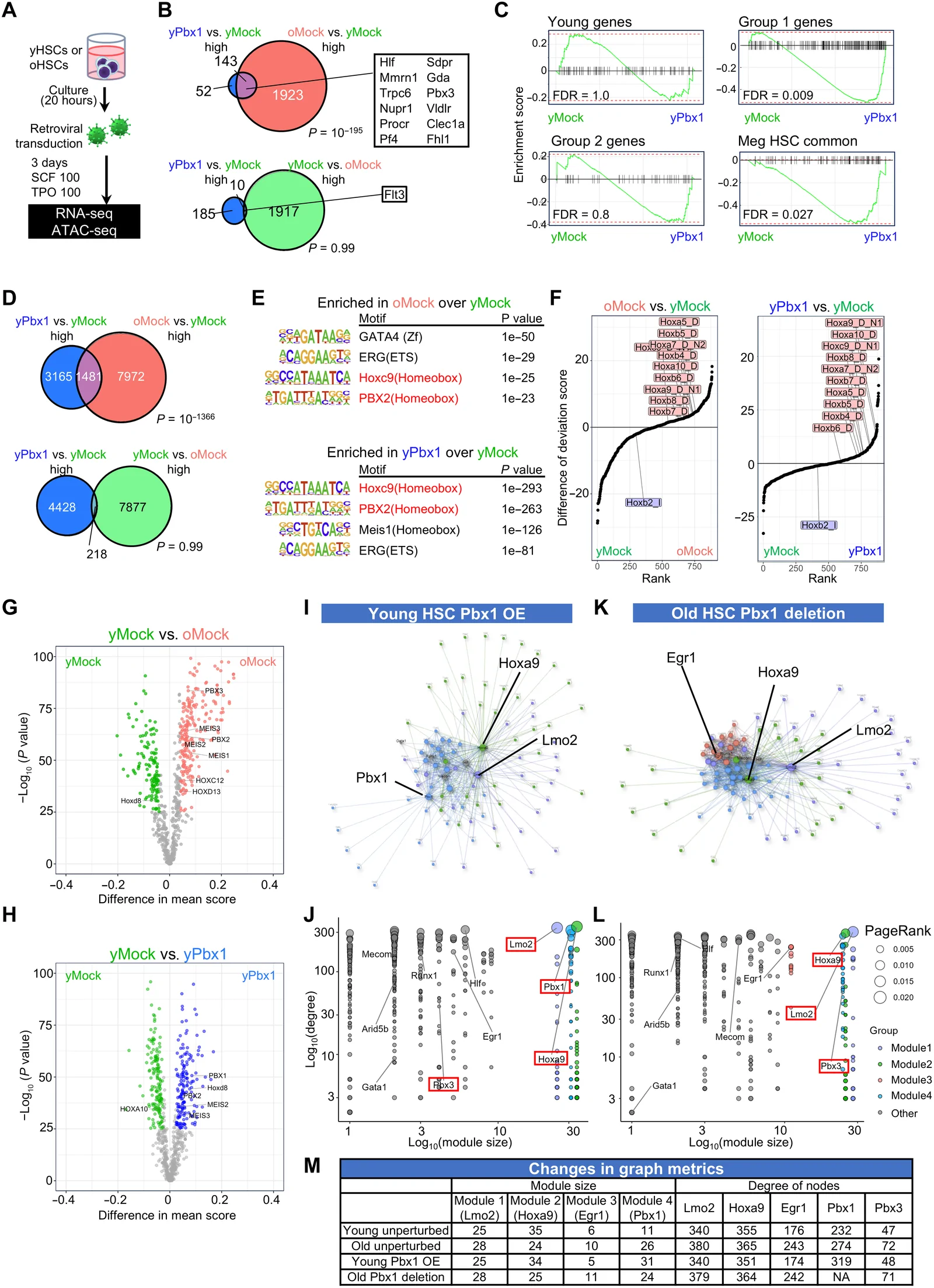
Pbx1 activates a core transcriptional network shared by aging and stemness. (**A**) Schematic of RNA-seq and ATAC-seq following Pbx1 overexpression. CD150^+^CD48^−^CD41^−^CD34^−^ LSK cells were isolated either from young mice (2.5 months; 4 mice pooled per sample) or from old mice (18 months; 1 mouse per sample). Thus, each sample comprised cells from four young mice or one old mouse. Following transduction, 5000 GFP^+^ cells were collected for RNA-seq and 50,000 GFP^+^ cells for ATAC-seq, and libraries were generated for each assay (n = 3 biological replicates). (**B**) Overlap between genes up-regulated in yPbx1 versus yMock and those up-regulated in oMock versus yMock (top), and between genes up-regulated in yPbx1 versus yMock and those up-regulated in yMock versus oMock (bottom). (**C**) GSEA comparing yPbx1 and yMock. (**D**) Overlap between DARs with increased accessibility in yPbx1 versus yMock and those increased in oMock versus yMock (top) and between DARs increased in yPbx1 versus yMock and those increased in yMock versus oMock (bottom). (**E**) HOMER-predicted motifs enriched in indicated groups. (**F**) Rank-ordered motif enrichment by chromVAR comparing oMock versus yMock (left) and yPbx1 versus yMock (right). (**G**) TOBIAS-predicted enriched TF footprints comparing yMock and oMock. (**H**) TOBIAS-predicted enriched TF footprints comparing yMock and yPbx1. (**I**) Network architecture of young HSCs following in silico Pbx1 overexpression; genes in large modules are shown (colored). (**J**) Network metrics for young HSCs following in silico Pbx1 overexpression, including degree, PageRank, and module size; modules with >9 genes are colored. (**K**) Network architecture of old HSCs following in silico Pbx1 deletion. (**L**) Network metrics for old HSCs following in silico Pbx1 deletion. (**M**) Summary of representative graph metrics (node count and module size) for the indicated genes. FDR, false discovery rate.

To investigate the transcriptional program induced by Pbx1 up-regulation, we performed RNA-seq and ATAC-seq simultaneously 3 days after Pbx1 transduction in HSCs (Fig. 5A). Although the number of Pbx1-regulated genes is smaller than the number of genes commonly up- or down-regulated in old HSCs (fig. S6A), and the shift toward the aging-associated axis (PC1) in the PCA plot was modest (fig. S6B), up to 73.3% of genes up-regulated in young Pbx1 HSCs (yPbx1) compared to young Mock HSCs (yMock) overlapped with genes up-regulated in old Mock HSCs (oMock) versus yMock HSCs (Fig. 5B). Conversely, genes up-regulated in yMock relative to yPbx1 significantly overlapped with genes expressed at higher levels in yMock than in oMock (fig. S6C). Genes commonly up-regulated in yPbx1 and oMock include HSC-related Hlf and Procr or megakaryocyte-related Pf4 (Fig. 5B and fig. S6, D and E). In contrast, few genes up-regulated in yPbx1 HSCs overlapped with genes up-regulated in yMock versus oMock, with the notable exception of Flt3 (Fig. 5B and fig. S6E). Genes down-regulated in yPbx1 HSCs included differentiation-related Cd34, Cd244, and Cd48, whereas some old genes up-regulated in oMock including Vwf and Slamf1 were down-regulated (fig. S6E). Gene set enrichment analysis (GSEA), together with analysis of overlapping gene sets, indicated that the global difference between young and old was maintained in both Mock- and Pbx1-transduced cells (fig. S6, C and F). In contrast, while neither young nor Group 2 old genes were significantly altered, Group 1 old genes were enriched in yPbx1 as well as in genes shared by young megakaryocytes and young HSCs, suggesting that Pbx1 regulates old genes shared by old HSCs and stem cell genes and partially megakaryocyte-related genes (Fig. 5C).

Similarly, although the number of DARs altered after Pbx1 transduction was small (fig. S7A), the shift toward the aging-associated axis (PC1) in the PCA plot was modest (fig. S7B), and up to 66% of age-related DARs were constant after Pbx1 transduction (fig. S7C). ATAC-seq revealed that 31.8% of DARs up-regulated in yPbx1 were shared with DARs higher in oMock than in yMock, whereas only 4.7% shared with yMock high regions (Fig. 5D). Conversely, DARs high in yMock versus yPbx1 and yMock versus oMock was exhibited significant overlap, whereas DARs high in yMock versus yPbx1 and oMock versus yMock showed little overlap (fig. S7C). The analysis of feature distribution on genomic region of DARs of each comparison among yMock, yPbx1, oMock, and oPbx1 did not exhibit obvious difference in contrast to fresh and short-term stimulation in Fig. 2, perhaps due to longer period of ex vivo culture (fig. S7D).

Motif analysis revealed that similar to short-term proliferative stimulation (Fig. 2), Homeobox motif was overrepresented in oMock and in yPbx1 compared with yMock (Fig. 5, E and F). As a Pbx1 partner, Meis1 motif (*71*) was also enriched in yPbx1 (Fig. 5E). TOBIAS footprint analysis also showed that Pbx motif and Hox motifs are overrepresented in either oMock group (Fig. 5G) or yPbx1 group (Fig. 5H), suggesting that Pbx1 is directly bound to open chromatin regions. Notably, Irf motif, which was enriched in steady-state old HSCs (Fig. 2E), and FoxO motif were highly enriched in oMock compared with yMock (fig. S7E), whereas these motifs were not enriched in yPbx1 (fig. S7E), suggesting that multiple mechanisms other than Pbx1 are responsible for old-specific epigenome formation.

Network analysis following in silico perturbation was conducted as described in Fig. 4G. The overexpression of Pbx1 in young HSCs led to an expansion of the Pbx1 module, resembling the native old HSC network, whereas Hoxa9 and Lmo2 modules remained unchanged (Figs. 4, H and I and 5I). In contrast, the deletion of Pbx1 in old HSCs resulted in contraction of the old HSC gene network (Figs. 4, J and K and 5, J and K). Notably, the extent of network contraction was relatively modest, which may be attributable to the intrinsic robustness of the age-associated GRN architecture once established, potentially in part due to compensatory activity by Pbx3 (Fig. 5, L and M).

The functional relationship between open chromatin and expression levels was examined using Genomic Regions Enrichment of Annotations Tool (GREAT) (*72*). Around genomic region with higher signals in yPbx1, old genes such as Nupr1 and Sdpr were located, which was also observed in oMock HSCs (fig. S7, F and G). Similarly, a subset of genes negatively regulated by Pbx1, including Mpo and Cd48, were associated with decreased accessibility (fig. S7, H and I).

To further relate accessibility changes to the RNA-seq signatures, we performed gene set–based comparisons of gene-level ATAC accessibility at promoters [TSS, ±500 base pairs (bp); fig. S7J] and putative enhancers (±10 kb of gene bodies; fig. S7K). Consistent with the broad age-associated shift observed in the oMock versus yMock comparison, Group 1, Group 2, MolO, and Meg HSC common gene sets showed significant enrichment at both promoters and putative enhancers. In contrast, following Pbx1 transduction (yPbx1 versus yMock), enrichment was more restricted. Group 1 and Meg HSC common gene sets were significantly enriched at putative enhancers, whereas only the Meg HSC common gene set reached significance at promoters, mirroring the GSEA results and supporting the notion that Pbx1 preferentially engages a subset of the age-associated GRN.

### Pbx1 is responsible for platelet bias in old HSCs

**Fig. 6.**
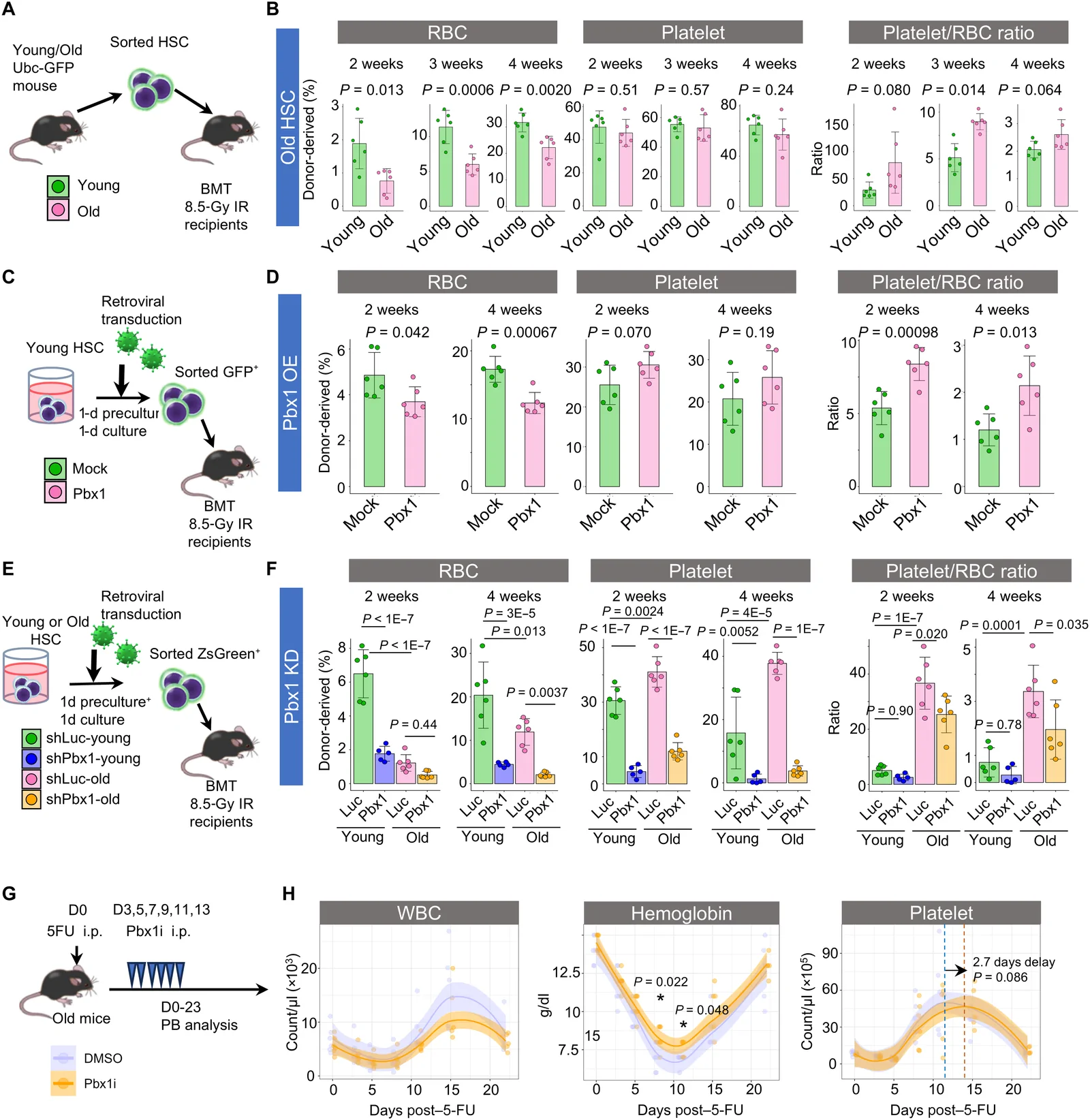
Pbx1 is responsible for platelet bias in old HSCs. (**A**) HSCs (CD150^+^CD48^−^CD41^−^ LSK cells for young and CD150^+^CD48^−^ LSK cells for old) were collected from Ubc-GFP mice. Each of 400 sorted GFP^+^ cells were transplanted to irradiated mice along with GFP^−^ competitor cells. (**B**) Frequency of donor-derived RBCs and platelets, and the platelet/RBC ratio in PB after BMT (means ± SD, *n* = 6 from a single experiment), unpaired two-tailed *t* test. (**C**) Mock or Pbx1 was retrovirally transduced in young HSCs and 1000 GFP^+^ cells 1 day after transduction were transplanted to irradiated mice. (**D**) Frequency of donor-derived RBCs and platelets, and the platelet/RBC ratio in PB after BMT (means ± SD, *n* = 6 from a single experiment), unpaired two-tailed *t* test. (**E**) Either shLuc or shPbx1 was retrovirally transduced in young or old HSCs and 1000 Zs-Green^+^ cells 1 day after transduction were transplanted into irradiated mice. (**F**) Frequency of donor-derived RBCs and platelets, and the platelet/RBC ratio in PB after BMT (means ± SD, *n* = 6 from a single experiment, Tukey’s multiple comparison test). (**G**) Old mice (19 m old) received 5-FU (150 mg/kg, i.p.), followed by administration of Pbx1 inhibitor (10 μg per mouse; Pbx1i) from day 3 to day 13, every other day. (**H**) White blood cell (WBC) count hemoglobin concentration and platelet count were examined at indicated time points. During the follow-up period, two mice in the Pbx1i group died (*n* = 6 for each group; shaded area indicates 95% confidence interval). *P* values for comparisons of count data were determined using an unpaired two-tailed *t* test. The *P* value for delayed platelet recovery was assessed using a log-rank test, and the peak difference was estimated using the survfit function. Dashed line on the Plt panel indicates the time point of the highest Plt count. KD, knockdown; OE, overexpression. IR, irradiation.

Next, we investigated the contribution of Pbx1 to aging-associated hematopoietic output and lineage bias in vivo. To establish an age-associated functional baseline, we transplanted freshly isolated HSCs from young or old Ubc-GFP donor mice into lethally irradiated recipients (Fig. 6, A and B). In this setting, early red blood cell (RBC) output (weeks 2 to 4) was reduced, and the platelet-to-RBC ratio (week 3) was increased in recipients of old HSCs, whereas platelet chimerism was comparable to that observed with young HSCs (Fig. 6B). By 2 months posttransplantation, total chimerism was decreased, platelet chimerism was lower than that in the young group, and the platelet bias was no longer evident (fig. S8, A and B).

To directly test the role of Pbx1 in these phenotypes, we perturbed Pbx1 expression in HSCs and evaluated early hematopoietic output after transplantation. To establish a baseline for aging-associated changes under viral transduction conditions, we also transplanted retrovirally GFP-transduced young and old HSCs (fig. S8C; transduction efficiency before sorting is summarized in table S7) and observed qualitatively similar trends, although viral marking and short-term culture increased variability and altered overall chimerism kinetics (fig. S8, D and E).

We then overexpressed Pbx1 in young HSCs and transplanted them into recipients (Fig. 6, C and D, and fig. S9, A to E). The retroviral overexpression of Pbx1 resulted in decreased regeneration of RBCs and a bias toward platelet production 2 weeks after transplantation (Fig. 6D and fig. S9D). The retroviral overexpression of Pbx1 decreased RBC reconstitution while preserving platelet output, thereby inducing a platelet-biased reconstitution pattern at 2 weeks posttransplantation (fig. S9, B and E). In contrast, retroviral knockdown of Pbx1 in old HSCs (Fig. 6, E and F, and fig. S10, A to E) reduced platelet bias (Fig. 6D and fig. S10D). However, this attenuation of platelet bias was accompanied by reduced RBC production in the Pbx1 knockdown group (fig. S10, B and E), potentially reflecting a requirement for precise temporal and dosage control of Pbx1 expression for optimal RBC production and HSC maintenance. Therefore, we investigated effects of short-term Pbx1 inhibition in old mice by administering a Pbx1 inhibitor (*73*) in vivo after bone marrow injury induced by 5-fluorouracil (5-FU) treatment (Fig. 6G). This intervention resulted in a modest decrease in the RBC nadir. While the delay of platelet recovery did not reach statistical significance, the peak timing showed a consistent shift across experiments, supporting a trend toward altered recovery kinetics (Fig. 6H). Together, these results suggest that short-term Pbx1 inhibition can modestly modulate post-injury hematopoietic recovery and may partially influence platelet bias in vivo.

To investigate the role of Pbx1 up-regulation in old HSC phenotypes, we searched for its targets in old HSCs. Among genes involved in megakaryocyte/erythroid lineage commitment, Gata1 and Pf4 were the most differentially expressed genes upon Pbx1 overexpression (fig. S11A). ATAC-seq analysis revealed that the Gata1 locus was more accessible in young HSCs even after 26 hours of stimulation, whereas the same Gata1 region was closed upon Pbx1 overexpression (fig. S11B), suggesting that Pbx1 regulates Gata1 expression. The expression level of Gata1 tended to be lower in old HSCs compared to young HSCs after 3 days of cytokine stimulation in vitro (fig. S11C). In addition, to evaluate effects of other TFs in comparison to Pbx1, we performed retroviral overexpression of various TFs in young HSCs, including those associated with self-renewal and early hematopoietic differentiation, and analyzed gene expression changes. Pbx1 overexpression consistently down-regulated Gata1 expression, whereas expression of the old HSC-related genes, Sdpr and Ehd3, was preserved (fig. S11D). In contrast, known regulators of megakaryocyte differentiation, such as small MAFs (Mafg and Mafk) (*74*) or Runx1 (*56*), up-regulated Gata1 expression. In support of this finding, the knockdown of Pbx1 using retroviral shRNA expression in HSCs resulted in up-regulation of Gata1 (fig. S11E), confirming the negative regulatory role of Pbx1 in Gata1 expression. When evaluating four publicly available unbiased scRNA-seq datasets focusing on immature populations in the bone marrow (fig. S11F), megakaryocytic progenitors (characterized by Pf4 expression) consistently showed remarkable up-regulation of Pbx1. In contrast, Pbx1 was not expressed in erythroid progenitors (characterized by Klf1 expression). In addition, Gata1 expression levels were significantly higher in erythroid progenitors compared to megakaryocytic progenitors (fig. S11F), as reported previously (*75*). Consistent with prior scRNA-seq datasets, repopulating young HSCs express negligible Gata1 transcripts, whereas Gata1 is rapidly induced only upon erythroid commitment (*76*, *77*). Notably, increased Pbx1 and Pf4 expression accompanied by reduced Gata1 expression was also observed in old megakaryocytes relative to their young counterparts, whereas these expression levels were not different in MEPs or erythroid cells (fig. S11G). These findings indicate that aging differentially affects erythroid versus megakaryocytic lineage trajectories. Collectively, our data support a model in which Pbx1 compromises the induction, rather than the basal expression, of Gata1 during early erythroid commitment, thereby biasing old HSCs toward platelet production at the expense of erythropoiesis.

## DISCUSSION

In this study, using optimized deep learning-based models, we demonstrated that the genetic network defining stem cell identity shares a core component with the aging-associated network, with Pbx1 serving an essential function in its regulation (fig. S12). Upon categorizing genes specifically up-regulated in old HSCs into two groups, one group common to the most primitive HSC genes (Group 1) and another common to young megakaryocytic progenitor genes (Group 2), Pbx1 predominantly regulates group 1 genes associated with old HSCs. Pbx1, a TF with a Three Amino Acid Loop Extension (TALE) homeodomain, functions through heterodimer formation with Hox proteins or other TALE domain proteins, such as Meis1 (*78*). It is essential for maintaining quiescent HSCs (*65*). It is also associated with thrombocythemia in myeloproliferative disorders (*79*).

Recent studies have reported that the transcriptional profile of old HSCs can partially revert to young state following transplantation into a young recipient environment (*30*, *80*). These findings indicate that extrinsic cues from the niche can transiently modulate the aged transcriptome. However, this rejuvenation is incomplete, as the functional bias toward platelet production and the diminished long-term repopulating capacity of old HSCs remain largely uncorrected.

Consistent with these observations, our results demonstrate that although certain young transcriptional features are restored under young conditions, the global gene regulatory network remains skewed toward an aging-associated configuration. This suggests that intrinsic transcriptional and epigenetic memory, mediated in part by regulators such as Pbx1, maintains the aged state even after environmental reprogramming.

Old HSCs exhibit a differentiation defect, reflecting a more dormant HSC phenotype (*81*). Consistent with this, old HSCs manifest lower demethylation status at the self-renewal–associated HOX gene locus, similar to HSCs with *Dnmt3a* mutations (*22*, *82*). Although clear clonal hematopoiesis of indeterminate potential (CHIP)-related gene mutations are not observed in old mice, the increase in HSCs with old phenotypes and enhanced self-renewal capacity plausibly leads to selection of cells with stem cell–like properties over their life course, similar if not identical, to those observed in other stem cells (*83*–*85*), and increased Pbx1 expression potentially confers resistance to selective pressure. However, this selective pressure theory does not account for the presence of several genes, such as Mllt3 (*86*) or Hoxb4 (*87*), which lead to an increase in HSCs when overexpressed, but which are not overrepresented in old HSCs. One possible explanation for this is that HSCs function as megakaryocyte progenitors under steady-state conditions (*88*, *89*) and have a continuous drive to up-regulate the megakaryocytic program, in part via TPO (*90*). Only a small number of transcription/epigenetic factors, including Pbx1, may lie at the intersection of the megakaryocyte and HSC programs. Consistent with this, recent reports have shown that CD48^−^ MkPs directly derived from either young or old HSCs (*6*, *12*, *91*) are responsible for thrombopoiesis, suggesting an intriguing relationship with the aging GRN controlled by Pbx1.

On the other hand, while Pbx1 certainly regulates old genes, it does not explain all age-related changes. It can account for enrichment of the Homeobox motif in the epigenome, but it cannot explain enrichment of IRF, AP-1, or FoxO target sequences observed after proliferative stimuli, characteristic of aging. This suggests that age-related changes result from complex, multifactorial processes. We have shown that Pbx1 is involved in suppression of Gata1 expression and erythroid output. However, the myeloid bias and reduced repopulation capacity observed in old HSCs were not linked to Pbx1 overexpression within the observation period of our primary transplantation assays. Although Pbx1 is included in our Group 2 gene set and is highest in the megakaryocytic lineage, our perturbation datasets indicate that Pbx1 preferentially reinforces the Group 1/HOX stemness module, with only modest early changes in the Group 2 transcript set (Fig. 5C). We therefore interpret enrichment of this CD41^+^ SLAM-LSK–derived Group 2 signature as a conservative marker of megakaryocytic/platelet priming in old HSCs that likely reflects additional regulators and/or kinetics beyond the immediate transcriptional effects of Pbx1. This framing is consistent with the platelet-biased output observed upon Pbx1 perturbation, while avoiding an overstatement that Pbx1 alone explains all aging-associated phenotypes (Fig. 6).

A notable aspect of this study is the function served by deep learning–based models in our biological findings. While it has been previously shown that BERT/transformer-based GRN models exhibit high classification performance across different cell types, this study demonstrates that fine-tuned BERT/transformer-based GRN models can accurately model subtle differences in GRNs related to age changes in rare stem cell populations in different species, both power and flexibility for broad applications. The network structure incorporated into the model revealed notable differences among TFs in their roles in transcriptional regulation. Certain TFs, such as Hlf and Mecom, exhibit high centrality within the network but are associated with small module sizes, indicating weak connections to a broad range of HSC-specific genes. In contrast, Pbx1 is characterized by larger module sizes and displays relatively strong associations with age-related genes, which may underlie the consistent expression of aging-associated HSC genes across individuals. Furthermore, in silico perturbation analyses demonstrated an asymmetric role for Pbx1 upon overexpression or deletion. Specifically, the overexpression of Pbx1 led to the formation of a large module in young HSCs, while the deletion of Pbx1 exerted a relatively modest impact on the GRN of old HSCs. This is experimentally supported by the fact that the effects of Pbx1 suppression or inhibition in oHSC are relatively mild compared to Pbx1 overexpression in yHSC (Fig. 6). These findings may account for the robust and unidirectional alteration observed in age-related transcriptional programs.

Although we identified Pbx1 by combining network analysis with attention rollout, inference from in silico perturbation, and functional screening of TFs, differentiation defects alone do not account for all aspects of HSC aging, suggesting the need to consider combining other features of aging. Factors such as Irf, AP-1, and FoxO that are reportedly critical for aging of hematopoietic and other stem cells (*92*–*94*) are regulated not only by TF expression levels but also by post-translational/extracellular mechanisms such as inflammation (*95*), niche-derived factors (*96*), replication stress (*21*), metabolic states, reactive oxygen species (*97*, *98*), and dysfunctional proteostasis (*99*, *100*). Learning from gene expression data alone may not adequately model higher-order architecture of bona fide GRNs. Incorporating broader intra- and extracellular information such as single-cell level epigenome data (*101*, *102*), proteomics, phosphoproteomics, and metabolomics may allow development of models that more accurately represent aging states. In addition, although this study used identical datasets to fine-tune three models, predictions varied somewhat depending on models and methods used. There is room for further investigation into the ideal architecture and training methods for GRNs, including aspects such as appropriate amounts of data, preprocessing, hyperparameter settings, and model size.

The identification of Pbx1 as a central integrator of age-associated transcriptional programs provides important insight into hematologic pathologies in older individuals. Given that a platelet-biased phenotype is also observed in old human HSCs (*5*), together with reports indicating that age-associated thrombopoiesis is linked to an increased thrombotic tendency (*6*), Pbx1 may contribute to platelet-related, age-associated cardiovascular events. Moreover, persistent HOX motif accessibility and limited chromatin remodeling at differentiation-associated sites in old HSCs suggest that the Pbx1-Hox axis reinforces a relatively rigid epigenetic state.

In hematologic malignancies, PBX1 was first identified through the t(1;19)-derived E2A-PBX1 fusion in pediatric pre-B acute lymphoblastic leukemia (*103*, *104*). Our findings suggest that, even in nonmalignant old HSCs, a Pbx1-centered program preserves primitive transcriptional features and that acquisition of old HSC-associated signatures begins early in life, with Group 1 rising prenatally and Group 2 priming emerging during the first weeks after birth (fig. S1I). The selective retention of these regulatory states despite functional decline may facilitate the acquisition of secondary mutations and provide a substrate for cooption during clonal hematopoiesis or leukemia progression. Collectively, this Pbx1-centered network links early-life transcriptional trajectories to physiological HSC aging and age-associated hematologic disease.

## MATERIALS AND METHODS

### Study design

In this study, we integrated cross-sectional profiling with large-scale single-cell data analysis using a pretrained GRN model, Geneformer, to investigate high-dimensional alterations in the GRN of young and old HSCs. This approach was complemented by interventional experiments, including gene perturbation, small-molecule inhibition, and bone marrow transplantation to identify key transcriptional and epigenetic drivers of HSC aging. Single-cell and bulk RNA-seq and ATAC-seq were performed on young (8 to 14 weeks) and old (18 to 28 months) HSCs to catalog age-dependent changes in gene expression, chromatin accessibility, and TF occupancy. The resulting multiomics datasets were integrated with the attention-based Geneformer model, which enabled the ranking of candidate hub regulators using the attention rollout method and in silico perturbation to predict network-wide effects. These predictions were subsequently validated by experimental assays. Through TF overexpression screens, Pbx1 was identified as the predominant age-associated regulator. Functional effects were quantified by retroviral knockdown of Pbx1 in old HSCs and overexpression in young HSCs, assessing differentiation in vitro (cytokine-stimulated cultures) and in vivo (competitive bone marrow transplantation, 5-FU challenge). Formal sample size calculations, randomization, or blinding were not applied. Group sizes and allocation were determined on the basis of animal availability and logistical considerations in accordance with established field standards.

### Mice

C57BL/6J or C57BL/6N mice (8 to 14 weeks for young mice and 18 to 28 months for old mice purchased from SLC Japan or CLEA Japan) were used in all experiments, unless otherwise stated. C57BL/6-Ly5.1 congenic mice purchased from CLEA Japan were used for competitive repopulation assays. Scl-tTA::H2b-GFP mice (*105*) were purchased from the Jackson laboratory and genotyped as described in table S8. Ubc-GFP mice were purchased from the Jackson laboratory. All mice were bred in the animal facility at Keio University or the National Center for Global Health and Medicine under specific pathogen–free conditions and fed ad libitum. At a concentration of 200 mg/liter, doxycycline (Nacalai Tesque) was mixed in drinking water for H2B-GFP withdrawal in Scl-tTA::H2b-GFP mice from 12 to 14.5 months. Mice were euthanized by cervical dislocation. Animal experiments were approved by Tohoku University (24-087), Keio University (08104-8), and the National Institute of Global Health and Medicine (2025-A013). Both male and female mice were used in experiments.

### Cell preparation

Mouse bone marrow cells were isolated from two femurs and tibiae, and in some experiments, pelvises were used. Femurs and tibiae (and pelvises) were flushed with phosphate-buffered saline (PBS) + 2% fetal bovine serum (FBS) using a 21-gauge needle (Terumo) and a 10-ml syringe (Terumo) to collect the bone marrow plug. The plug was dispersed by refluxing through the needle and the suspension centrifuged at 680*g* for 5 min at 4°C. Cells were then lysed with lysis buffer (0.17 M NH_4_Cl, 1 mM EDTA, and 10 mM NaHCO_3_) at room temperature for 5 min, washed with 2 volumes of PBS + 2% FBS, and centrifuged at 680*g* for 5 min at 4°C. Cells were resuspended in PBS + 2% FBS and filtered through 40-μm nylon mesh (BD Biosciences). Cells were again centrifuged 680*g* for 5 min at 4°C and treated with anti-CD16/32 antibody for Fc-receptor block (2 μl per mouse) for 5 min at 4°C. In some experiments, anti–c-Kit magnetic beads (Miltenyi) were mixed at a 1:5 v/v ratio for 15 min at 4°C to concentrate HSPCs. After removing the antibody with two PBS + 2% FBS washes, c-Kit–positive cells were isolated using Auto-MACS Pro (Miltenyi) using the Possel-S program or Possel-d2 program. Isolated cells were centrifuged once at 340*g* for 5 min and stained with antibodies for flow cytometry.

### Cell culture

SF-O3 medium (Iwai North America) supplemented with recombinant human insulin, recombinant human transferrin, sodium selenite, ethanolamine, Hepes, sodium bicarbonate (2.2 g/liter) (supplied together with SF-O3), and 0.1% (v/v) bovine serum albumin (BSA) (Merck) was used. After dissolving all components into distilled water, the SF-O3 medium was saturated with CO_2_ generated from dry-ice by passing it through the medium. Sorted cells were centrifuged at 340*g* for 5 min, and supernatants were discarded. Medium was added to dilute cells to the desired number of cells per well in 10 μl, which was dispensed into 200 μl of culture media. Unless otherwise stated, SCF (100 ng/ml) and TPO (100 ng/ml) were supplemented, and culture conditions were 20% O_2_ + 5% CO_2_ at 37°C in appropriate humidified incubators. For RNA-seq and ATAC-seq data in Fig. 2, either 3000 or 30,000 HSCs were cultured in SF-O3 + 4% (w/v) BSA with SCF (1 ng/ml) and TPO (100 ng/ml) cultured for 26 hours in 1% O_2_ + 5% CO_2_, under conditions that promote proliferation while maintaining HSCs (*106*).

### Flow cytometry and cell sorting

Experiments were analyzed as described before (*106*) with modification. After selection of c-Kit^+^ cells using magnetic beads, the HSPC fraction was labeled as follows. For staining of C57BL/6 mice, lineage marker (CD4, CD8a, Gr-1, Mac-1, Ter-119, and B220)-peridinin–chlorophyll–protein complex (PerCP)-Cy5.5, c-Kit–allophycocyanin (APC)–Cy7, Sca-1–phycoerythrin (PE)–Cy7, CD150-PE, CD41–fluorescein isothiocyanate (FITC)/CD41-APC, CD48-FITC, Flt3-APC, CD34-BV421 were used. All antibodies used were 0.5 μl per mouse. Cells were resuspended in 0.5 to 2 ml of PBS + 2% FBS + 0.1% propidium iodide (PI) and sorted by FACSAria II or FACSAria IIIu into SF-O3 containing 0.1% (w/v) BSA. HSCs were defined as CD150^+^CD41^−^CD48^−^CD34^−^ LSK cells or CD150^+^CD41^−^CD48^−^LSK (*62*) or CD150^+^CD48^−^LSK cells unless otherwise stated. Data were analyzed using FlowJo software (BD Biosciences).

### MACSQuant analysis of cell number

Experiments were analyzed as described before (*106*). Most (170 μl) of the medium in wells of a 96-well plate was aspirated and samples were stained with 10 μl of antibody cocktail for 30 min at 4°C. Antibodies used were anti-lineage markers (CD4, CD8a, Gr-1, Mac-1, B220, Ter-119)-PerCP-Cy5.5, anti–c-Kit–APC–Cy7, anti–Sca-1–PE–Cy7, anti-CD150–PE, anti-CD48–FITC, anti-CD41–APC, and anti–P-selectin–BV421. All antibodies used were 0.1 μl per well. After incubation, 100 μl of PBS + 2% FBS was added to wells, and the plates were centrifuged 5 min at 4°C at 400*g* with low acceleration and medium deceleration. A 100 μl of supernatant was aspirated, and the cell pellet was resuspended in 200 μl of PBS + 2% FBS + 0.1% PI + 1% Flow-Check Fluorspheres (Beckman Coulter). Samples were acquired in fast mode, and volumes of 100 μL were analyzed. Data were exported as FCS files and analyzed using FlowJo software. Cell number was corrected by bead count of Flow-Check (∼1000/μl). Megakaryocytes were identified as cells with high forward scatter and side scatter and high CD150 and CD41 expression.

### Bone marrow transplant

Procedures are followed as previously described (*106*). For donor cells, HSCs (CD150^+^CD41^−^CD48^−^CD34^−^ LSKs) from C57BL/6-Ly5.2 mice or retrovirally transduced GFP^+^ cells or HSCs from Ubc-GFP mice, together with 8 × 10^5^ BMMNCs (for Ubc-GFP HSC transplant) or 4 × 10^5^ BMMNCs from untreated C57BL/6-Ly5.1 or Ly5.2 mice, were used. For recipients, C57BL/6-Ly5.1 or Ly5.2 congenic mice were used. Donor cells were transplanted retro-orbitally into recipients that had been lethally irradiated [8.5 gray (Gy) for Ubc-GFP HSC transplants or 9.5 Gy using MBR-1520R (Hitachi Power Solutions), 125 kV, 10 mA, 0.5-mm Al, 0.2-mm Cu filter]. After bone marrow transplantation (BMT) at indicated time points, peripheral blood was collected, and the percentage of donor-derived cells and their differentiation status were determined by FACS Aria or MACSQuant. Up to 40 μl of peripheral blood was sampled from the retro-orbital plexus using heparinized glass capillary tubes (Drummond Scientific) and was suspended in 1 ml of PBS + 10 U of heparin. For platelet and RBC analysis, 5 μl of blood suspension was mixed with 50 μl of staining solution [PBS + 2% FBS with Ter-119–PerCP–Cy5.5 (1:100) and anti–CD41-PE (1:100)] for 30 min at 4°C, washed once, and analyzed by MACSQuant. For white blood cell analysis, the blood suspension was centrifuged at 340*g* for 3 min. The supernatant was discarded, and the pellet was resuspended into 1 ml of PBS + 1.2% (w/v) dextran (200 kDa; Nacalai Tesque) for 45 min at room temperature. The supernatant then was centrifuged at 340*g* for 3 min, and pellets were resuspended in 0.17 M NH_4_Cl solution to lyse residual RBCs for 5 to 10 min until the suspension became clear. Cells were resuspended in 50 μl of PBS + 2% FBS with 0.3 μl of Fc-block. Surface antigen staining was performed using the following antibody panel: Gr1-PE-Cy7, Mac-1–PE–Cy7, B220-APC, CD4-PerCP-Cy5.5, CD8a-PerCP-Cy5.5, CD45.1-PE, and CD45.2-FITC. All antibodies were 0.3 μl per sample. The frequency (%) of donor-derived cells was calculated as follows:

100 × Donor-derived GFP^+^ cells (%)/[Donor-derived cells (%) + Competitor- or recipient-derived (Ly5.2^−^Ly5.1^+^ or GFP^−^) cells (%)]

Myeloid cells, B cells, T cells, RBCs, and platelets were marked by Gr-1^+^ or Mac-1^+^, B220^+^, CD4^+^ or CD8^+^, CD41^−^ Ter119^+^, and CD41^+^ Ter119^−^, respectively. The total cell chimerism represents the frequency of donor-derived GFP^+^ cells over the frequency of GFP^−^ cells in mononuclear or unlysed cells.

### Retroviral transduction for overexpression

cDNA encoding genes listed in table S4 were subcloned into the pMYs–internal ribosomal entry site (IRES)–enhanced GFP (EGFP) vector or into the pGCDNSam-IRES-EGFP vector for overexpression (*63*). Plasmids were transfected into Plat-E packaging cells using FuGENE HD (Promega) at a ratio of 1:2.5 mixed in Opti-MEM (Invitrogen) at room temperature for 15 min. Transfected cells were incubated for an additional 2 days in Dulbecco’s modified Eagle’s medium (DMEM) supplemented with 10% FBS under 5% CO_2_. Supernatant containing retroviruses was filtered with a 0.45-μm filter membrane (Millipore) and subsequently used for transduction. HSCs for transduction were sorted 1 day before the procedure and precultured in 96-well fibronectin-coated or tissue culture U-bottom plates with SF-O3 under conditions of 20% O_2_ and 5% CO_2_ for 16 to 24 hours. Subsequent to the removal of the HSC culture medium, cells were resuspended in 20 μl per well of freshly prepared medium and transferred to either fibronectin-coated 96-well U-bottom plates (Iwaki) or nontreated 96-well plates coated with 50 to 100 μl of RetroNectin (25 μg/ml; TaKaRa Bio). They were then incubated with 200 μl of virus supernatant for 30 min at 37°C in a 5% CO_2_ atmosphere. CombiMag (OZ Biosciences) was used to facilitate viral transduction. Following this, the virus supernatant was replaced with SF-O3 for subsequent analyses.

### Retroviral transduction for knockdown

Double strand DNA coding shRNA targeting genes listed in table S8 were inserted into the pSIREN-RetroQ-ZsGreen vector (TaKaRa Bio). The transduction procedure is the same as retroviral transduction for overexpression.

### Western blotting analysis

A total of 50,000 CD150^+^ LSK cells were sorted, precultured for 20 hours, and distributed into four wells for transduction. Cells were transduced with Pbx1 or an empty (mock) vector or with knockdown vectors expressing luciferase (control) or shPbx1, as described above. After 3 days, GFP-positive (overexpression) or ZsGreen-positive cells were sorted, washed once with PBS, and lysed in radioimmunoprecipitation assay buffer. Proteins were resolved by SDS–polyacrylamide gel electrophoresis (30 mA, 30 min), transferred to polyvinylidene difluoride membranes (100 V, 60 min), blocked with 5% skim milk in PBS-T (0.1% Tween 20) for 1 hour at room temperature, and incubated overnight at 4°C with anti-Pbx1 (1:1000; rabbit mAb, #4342S, Cell Signaling Technology) diluted in Can Get Signal Solution 1 (Toyobo). After washing with PBS-T, membranes were incubated with horseradish peroxidase (HRP)–conjugated anti-rabbit immunoglobulin G (IgG) (1:1000; #7074S, Cell Signaling Technology) in Can Get Signal Solution 2 for 1 hour at room temperature. Bands were detected using Immobilon Western Chemiluminescent HRP Substrate (Millipore) and imaged with an LAS4010 system (GE Healthcare). For reprobing, membranes were stripped (Restore PLUS; Thermo Fisher Scientific), reblocked, and sequentially incubated with anti–β-tubulin (1:2000; mouse mAb, #86298S, Cell Signaling Technology) and HRP-conjugated anti-mouse IgG (1:2000; #NA9310V, GE Healthcare), followed by chemiluminescent detection.

### Screening of TFs down-regulating CD48

Retrovirus production and HSC preculture conditions were prepared as previously described. Plat-E packaging cells were seeded in a 96-well flat-bottom plate at a density of 5 × 10^4^ cells per well in 100 μl of DMEM supplemented with 10% FBS, and 8 hours postseeding, transfection was performed using 0.17 μg of plasmid DNA combined with 8.4 μl of OPTI-MEM and 0.42 μl of FuGENE HD per well. Each plasmid transfection was executed in duplicate. On the day designated for transduction, viral supernatant collected from two wells, containing the same plasmid, was pooled to achieve a final volume of 140 μl. This was then mixed with 9.17 μl of Opti-MEM and 0.83 μl of CombiMag before being used for HSC transduction in fibronectin-coated 96-well U-bottom plates. Virus supernatant was replaced with SF-O3 + SCF (100 ng/ml) and TPO (100 ng/ml) after 30 min of transduction, and transduced HSCs were cultured for another 7 days and surface markers and cell numbers of either GFP^+^ and GFP^−^ cells were examined using MACSQuant. Both mock controls and nontransduced controls were included in each 96-well plate.

### 5-FU administration and Pbx1 inhibitor treatment

Old mice were administered 5-fluorouracil (5-FU, Kyowa Kirin) intraperitoneally at a dose of 150 mg/kg with a 25-gauge needle on day 0. The Pbx1 inhibitor T417 (*73*) (ProbeChem) was prepared in a 1:1 PBS/dimethyl sulfoxide solution at a concentration of 100 μg/ml and administered intraperitoneally at a dose of 10 μg per mouse, six times on alternating days from day 3 to day 13. Peripheral blood counts were assessed from day 0 to day 23 using the Celltac α (Nihon Kohden).

### cDNA synthesis and quantitative RT-PCR

Experiments were analyzed as described before (*106*). The total RNA was isolated from ∼1000 to 15,000 freshly isolated or cultured cells per condition using the RNeasy Mini Kit (QIAGEN). First-strand cDNA was generated with SuperScript VILO (Thermo Fisher Scientific) in a 20-μl reaction volume following the manufacturer’s instructions. The qPCR was carried out with SYBR Premix Ex Taq II (TaKaRa Bio) according to the manufacturer’s instructions. The reaction mix consisted of 45 μl of SYBR Premix, 0.36 μl each of forward and reverse primers, 1.8 μl of ROX II dye, 41.48 μl of nuclease-free water, and 1 μl of cDNA; aliquots (20 μl) were distributed into four wells of a PCR plate. Amplification and fluorescence detection were performed on an ABI 7500 Fast Real-Time PCR System (Applied Biosystems) using the following cycling parameters: 95°C for 10 s, followed by 40 cycles of 95°C for 5 s, and 60°C for 34 s. Relative transcript abundance was calculated as 2^−(Ct − mean Ct of Capdh or Actb)^ and, unless otherwise indicated, values were normalized to the corresponding control condition.

### scRNA-seq analysis

Experiments were performed as described before (*107*) with modification. Fresh CD150^+^CD48^−^CD41^−^CD34^−^ LSK cells (HSCs) or CD150^+^CD48^−^CD41^+^ LSK (CD41^+^ SLAM-LSK) cells from 10-week-old mice or CD150^+^CD48^−^CD41^−^CD34^−^ LSK cells from 7-month-old, 12-month-old, 18-month-old, or 2-year-old were sorted into 500 μl of SF-O3. H2B single-cell cDNA synthesis was performed with a C1 Single-Cell Auto Prep System (Fluidigm). Reverse transcription and preamplification were performed according to the manufacturer’s instructions using the SMARTer Ultra Low RNA Kit for the Fluidigm C1 System (Clontech) and the C1 Single-Cell Auto Prep Reagent Kit for mRNA Seq (Fluidigm). For RNA controls, tubes #1, #4, and #7 of ArrayControl RNA Spikes (Thermo Fisher Scientific) were mixed at a ratio of 100:10:1, and the RNA Spike mixture was diluted 1:10,000 in C1 Loading Reagent (Fluidigm). Sorted HSCs were diluted in Suspension Reagent to ∼1000 cells/μl and loaded into a C1-Single-Cell Auto Prep IFC for mRNA-seq (5 to 10 μm). After loading cells, the integrated fluidic circuit (IFC) was observed microscopically using an INCell Analyzer 6000 (GE Healthcare) to ensure that single cells were captured in each well. Wells with 0, >1, or shrunken cells were excluded. A total of 79 wells for 2.5-month-old HSCs and 85 wells for 7-month-old HSCs, 70 wells for 12-month-old HSCs, 79 wells of 18-month-old HSCs, 53 wells of 24-month-old HSCs, 69 wells of H2B-GFP-high HSCs, 80 wells of H2B-GFP-low HSCs (sum of 2 replicates), and 96 wells of CD41^+^ SLAM-LSK cells (sum of two replicates) were considered valid. Thermal cycling conditions for cell lysis, reverse transcription, and preamplification were as follows: 72°C for 3 min, 4°C for 10 min, and 25°C for 10 min for cell lysis; 42°C for 90 min and 70°C for 10 min for reverse transcription; 95°C for 1 min and 5 cycles at 95°C for 20 s, 58°C for 4 min, and 68°C for 6 min, 9 cycles at 95°C for 20 s, 64°C for 30 s, and 68°C for 6 min, 7 cycles at 95°C for 30 s, 64°C for 30 s, and 68°C for 7 min, and 1 cycle at 72°C for 10 min for preamplification; and holding at 4°C until harvest. Amplified cDNA (∼3.5 μl) from each well was diluted with 10 μl of C1 DNA Dilution Reagent (Fluidigm) and frozen at −30°C until use. The sample in each well was further diluted 1:2 with C1 Harvest Reagent and subjected to tagmentation and Illumina sequencing library preparation using the Nextera XT DNA Sample Preparation Kit (Illumina) and a Nextera XT DNA Sample Preparation Index Kit (96 indices, Illumina). Then, 2.5 μl of tagment DNA buffer, 1.25 μl of amplification tagment mix, and 1.25 μl of the diluted cDNA sample were mixed and incubated at 55°C for 10 min in a thermal cycler. The tagmentation reaction was then stopped by adding 1.25 μl of neutralizing buffer (NT buffer). Next, samples were subjected to PCR amplification. A 6.25-μl aliquot of each sample was mixed with Nextera PCR Master Mix (NPM) buffer and 1.25 μl each of index primer 1 and index primer 2. Thermal cycling conditions were as follows: 72°C for 3 min, 95°C for 30 s, 12 cycles at 95°C for 10 s, 55°C for 30 s, 72°C for 1 min, and 1 cycle at 72°C for 5 min. A 1-μl aliquot from each well of amplified DNA was processed by two rounds of mixing and cleanup with AMPure XP beads (Beckman Coulter), and DNA was then eluted with 144 μl of DNA suspension buffer. Sequencing was performed using an Illumina HiSeq 2000 system. Sequence reads were aligned to the murine genome mm10 using Cufflinks version 2.1.1. Downstream analysis was performed by R package Seurat (version 4) (*108*). Cells with nFeature_RNA > 2000 and nCount_RNA > 100,000 were selected, and then top 2000 genes with highest variance using vst method were chosen for subsequent dimensional reduction with PCA. Elbow plot was used to determine the dimension to use for uniform manifold approximation and projection (UMAP) as 1 to 8. Clustering was performed with resolution 0.7, and the cluster was annotated from the predominant origin of cells in each cluster. AUCell scores were calculated using the AUCell package (*109*) with gene sets obtained from MSigDB (www.gsea-msigdb.org/gsea/msigdb) or custom datasets (*106*) (table S2). Cell cycle genes (S and G_2_-M phases) were obtained from the GSEABase package. Gene set variation analysis (GSVA) was performed using GSVA package with default parameters (*110*). Transcriptional divergence across aging stages was evaluated by a kBET mixing analysis (*45*) and LISI (*46*) neighborhood mixing analysis from multiple age groups. GO analysis was performed using enrichGO function from the clusterProfiler package and clustered and visualized the similar GO terms using emapplot function followed by pairwise_termsim function for clustering (*111*). WGCNA (*70*) was performed to construct and visualize the correlation network of HSC genes, aging-associated genes, and megakaryocytic genes as provided in table S6 using scRNA-seq data, as depicted in Fig. 1.

### Reanalysis of deposited scRNA-seq data

scRNA-seq datasets of young and old HSPCs generated using either SMART-based technology, consistent with the approach used in this study, or droplet-based platforms were compiled, and cells originally sorted as young or old HSCs were reanalyzed (GSE59114 for C57BL/6; GSE59114 for DBA; GSE70657 for dataset 2; GSE132357, GSE147729, GSE166709, and GSE175702 for dataset 3; and GSE166709, GSE175702, GSE255019, GSE107727, GSE132357, and GSE147729 for the data on fig. S11G). Raw count matrices were processed using the Seurat workflow and integrated with the SCTransform method (v2 flavor). Integration anchors were identified using reciprocal PCA (rpca). PCA, UMAP, and AUCell scoring were then performed for downstream clustering and visualization as described above. Young-HSC, Old-HSC, and CD41^+^ populations were defined on the basis of the original cell annotations, the age of the donor mice, and expression of the megakaryocyte marker Pf4.

### Bulk RNA-seq analysis

For RNA-seq data in Fig. 2, 3000 cells from either fresh or 26-hour cultured young/old HSCs were directly sorted into 700 μl (Fig. 2) of RLT buffer from the RNeasy kit plus 1% (v/v) 2-ME and stored at −80°C until use. The total RNA was extracted using the RNeasy Plus Micro Kit (QIAGEN), and the cDNA was synthesized using a SMART-Seq v4 Ultra Low Input RNA Kit for Sequencing (Clontech, Palo Alto, CA, USA). Double-stranded cDNA was fragmented using a S220 Focused-ultrasonicator (Covaris), and cDNA libraries were generated using the NEBNext Ultra DNA Library Prep Kit (New England BioLabs, Beverly, MA, USA). Sequencing was performed using HiSeq1500 (Illumina) with a single-read sequencing length of 60 bp. TopHat was used for mapping to the reference genome (UCSC/mm10). For data in Fig. 5, RNA from 5000 Mock- or Pbx1-transduced GFP^+^ cells was subjected to cDNA synthesis using the SMART-Seq HT Kit (Clontech, Palo Alto, CA, USA). Libraries were prepared using Nextera XT DNA Library Prep Kits with Nextera XT index Kit v2 primers. Sequencing was performed using a NovaSeq 6000 (Illumina) with a paired-end sequencing length of 150 bp. STAR (version 2.7.10) was used for mapping to the reference genome (UCSC/mm10) (*112*). Gene expression levels were quantified using featureCounts (*113*). Downstream analyses were performed using R (v4.3.0) programs (*114*). Count data were normalized and analyzed using DESeq2 (v1.30.1) (*115*) to detect differentially expressed genes. PCA was performed using the prcomp function for 500 genes with the highest variation among samples after transforming the raw count data using the vst function from the DESeq2 package. GSEA was performed using the fGSEA (v1.16.0) package (*116*) with the rank statistics according to the log fold changes of the DESeq2 results. Gene sets subjected to fGSEA were downloaded from MsigDB. Custom gene sets were generated to investigate HSPC-related gene set enrichment.

### ATAC-seq analysis

ATAC-seq library preparation for Fig. 2 was performed on 30,000 cells with Nextera DNA Sample Preparation kits (FC-121-1030, Illumina) according to a previously described protocol (*53*) and sequenced with a HiSeq1500 with a single-read sequencing length of 60 bp. For Fig. 5, 50,000 Mock- or Pbx1 transduced young or old HSCs were subjected to the Zymo-Seq ATAC Library Kit (Zymo Research) according to the manufacture’s instructions, and sequencing was performed using a NovaSeq 6000 with a paired-end sequencing length of 150 bp. Fastq files were trimmed using TrimGalore (version 0.6.7; DOI: https://doi.org/10.5281/zenodo.5127899) and aligned to the reference mouse genome (mm10) using Bowtie2 (*117*) with a --very-sensitive and single-read mode. Mitochondrial genomes, Y chromosomes, and scaffold chromosomes were removed using samtools, and duplicate reads were removed using picard MarkDuplicates. Peak calling was performed using MACS3 (*118*) for each group with -*q* = 0.05, --extsize =100, and --shift = 50 with narrow peak mode. All peaks called in each group were merged, and peaks overlapping with the blacklist region (*119*) were excluded. Peaks within a distance smaller than 50 bp were considered identical. After further removing peaks with average of log2 counts per million (logCPM) < −1, peak count around ±25 bp of each summit was performed using csaw package of R (*120*). PCA was performed using the prcomp function of R, following normalization based on the total counts for each sample. DARs between two groups were identified using edgeR (*121*) with false discovery rate < 0.05 considered as DARs. The findMotifsGenome.pl function from the HOMER software (*54*) was used to assess motif enrichment by comparing the DARs across the indicated groups. In addition, the matchMotifs function from motifmatchr package and the computeDeviations function from the chromVAR package (*59*), together with the mouse_pwms_v2 motif dataset from chromVARmotifs (*59*), were used to calculate differences in motif enrichment scores between the two groups. After aggregation of every sample in the same experimental group, the TOBIAS BINDetect program (*122*) was applied to determine exact cut sites from ATAC-seq data, following nucleotide bias correction with ATACorrect. The JASPAR2018_CORE_vertebrates_redundant.meme dataset (*123*, *124*) served as the motif reference. For TOBIAS analysis, we applied two thresholds: (i) an adjusted *P* value (*P*adj) < 1 × 10^−25^ to ensure strong statistical confidence after multiple-testing correction, and (ii) an absolute difference in mean score > 0.2. The GREAT web tool (*72*) was used to identify associated genes for each DAR. For gene set–level evaluation of chromatin accessibility, ATAC-seq peaks were assigned to genes and aggregated into gene-level accessibility scores using normalized accessibility (logCPM). Peaks were grouped as promoter-proximal (TSS, ±500 bp) or putative enhancers (within ±10 kb of gene bodies), and peak-level signals were summed per gene. For each predefined gene set, differences in gene-level accessibility distributions between conditions were assessed using a two-sided unpaired Wilcoxon rank-sum test.

### Visualization of ATAC

Preprocessed BAM files from each group were combined, and BigWig files were generated using the bamCoverage function from DeepTools with CPM normalization (*125*). To reduce the impact of background noise, scale factors were applied to normalize the total read counts mapped to identified peaks for each group. The computeMatrix function, followed by the plotProfile function, was used on BigWig files to create line plots representing aggregate peaks of young, Group 1 old, and Group 2 old genes. BigWig files were visualized using the Integrative Genomics Viewer (*126*).

### Relationship between Pbx1 knockout transcriptome and old genes

Differentially expressed genes in Pbx1 knockout HSCs compared with wild-type HSCs were obtained from (*65*). Overlapping genes either significantly up-regulated or down-regulated in knockout HSCs with old HSC genes of each indicated dataset obtained from Comprehensive Aging Signature of HSCs (https://agingsignature.webhosting.rug.nl/) or our scRNA-seq data in Fig. 1 were counted, and hypergeometric test was performed to calculate *P* values.

### Data preprocessing for transfer learning

scRNA-seq datasets analyzed for immature murine bone marrow cells were collected, which include experiments performed by both the droplet-based method and the SMART-based method. Raw count data were subjected to Seurat with parameters and normalization and integration methods described in table S9. For two-step fine-tuning, datasets consisting only of cells from young animals (GSE107727 and GSE147729 for young samples and GSE132357 and GSE175702 for young samples) were prepared for the task of classifying 13 cell types (HSC, ST-HSC, MPP, megakaryocyte-erythroid progenitor, MkP, erythroid, common lymphoid progenitor, B cell, granulocyte-macrophage progenitor, monocyte (Mono), dendritic cell, neutrophil (Neut), and mast cells (Mast). Clusters estimated by the FindClusters function of Seurat were manually assigned to one of the aforementioned cell types except for minor populations including, ILC2, plasma cells, and natural killer cells, which were ignored in the fine-tuning process, due to the low frequency in each dataset based on markers identified by the FindAllMarkers function. Datasets consisting of both young and old HSPC data (GSE59114, GSE59114-DBA, GSE166709, GSE175702-aged, GSE255019, and the data in Fig. 1) were prepared for the task of classifying 10 cell types (young HSC, old HSC, young ST-HSC, old ST-HSC, young MPP, old MPP, young MkP, old MkP, young other cell type, and old other cell type) based on either marker genes or the age of the source animal. For one-step fine-tuning, datasets consisting of only young cells were relabeled with five cell types (young HSC, young ST-HSC, young MPP, young MkP, and young other cell type). After annotation, gene IDs were converted to human Ensembl ID using the “getLDS” function of the biomaRt package of R with the December 2021 or May 2021 versions of the Ensembl archive and converted to dgCMatrix data, followed by conversion to the loom format with the loompy.create function of the loompy package of Python, as input for fine-tuning.

### Geneformer fine-tuning and in silico perturbation

Geneformer pretrained models [GF-6L-30M-i2048 (*34*) and GF-12L-95M-i4096 (*60*)] and associated Jupyter notebooks for fine-tuning, embedding visualization, and in silico perturbation were obtained from the Geneformer repository hosted on Hugging Face (*34*). The analyses were performed using the following repository revisions: commit 6caf4809f8cffc35569d896609228d0018789736 for the six-layer two-step model (https://huggingface.co/ctheodoris/Geneformer/commit/6caf4809f8cffc35569d896609228d0018789736) and commit c81f6f912c7e71483b9ca9560024904b3891f0bf for the six-layer one-step and 12-layer one-step models (https://huggingface.co/ctheodoris/Geneformer/tree/c81f6f912c7e71483b9ca9560024904b3891f0bf). The Geneformer model used for cell classification is based on the BertForSequenceClassification architecture. The GF-6 L-30 M-i2048 model comprises six transformer layers with four attention heads and supports an input gene size of 2048. The GF-12L-95M-i4096 model consists of 12 transformer layers with eight attention heads and an input gene size of 4096. Input scRNA-seq data in the loom format were tokenized using the tokenize_data function applied to the TranscriptomeTokenizer object within the Geneformer library. We developed three fine-tuned models: (i) training GF-6 L-30 M-i2048 with 10 datasets containing 160,005 cells using an 80% training and 20% evaluation split for a one-step classification task into 10 cell types (six-layer one-step model), (ii) initially training GF-6L-30M-i2048 with four datasets containing only young cells (112,124 cells, 80% training, and 20% evaluation) for a task classifying 13 cell types, followed by additional fine-tuning with six datasets that included both young and old cells (40,382 cells, 80% training, and 20% evaluation) to classify 10 cell types (six-layer two-step model), and (iii) training GF-12L-95M-i4096 with 10 datasets (160,005 cells, 70% training, 15% validation, and 15% testing) for a one-step classification task into 10 cell types (12-layer one-step model). AdamW was used as the optimizer for all models. Minimal learning epochs were selected to maximize the macro *F*_1_ score to prevent overfitting. Additional training hyperparameters are provided in table S10. The training process was executed using a single NVIDIA A100 40GB or 80GB GPU.

### Evaluation of the Geneformer model

The F1 score was determined using the f1_score function from the sklearn.metrics package in Python, with predicted and actual cell types serving as inputs. The receiver operating characteristic (ROC) curve and the area under the curve (AUC) were computed using the roc_curve and auc functions. Heatmaps depicting the output logit scores of the final layer and the confusion matrix were generated using the heatmap function from the seaborn package. After evaluation, a six-layer one-step model and a 12-layer one-step model were comparable in terms of *F*_1_ scores and accuracy distinguishing young and old HSCs, whereas a six-layer two-step model was inferior. Considering the computational resource requirements, the smaller six-layer one-step model was selected as the optimal model for subsequent downstream analyses.

### In silico perturbation

For in silico perturbation, either the “delete” or “overexpress” perturbation types were used. An InSilicoPerturber object from the Geneformer package was instantiated, specifying a randomly selected subset of 400 cells of the “cell type of interest” for perturbation, using the final layer for embedding (emb_layer = 0), and setting the forward_batch_size to 100. To avoid annotation bias, cells that were classified as a different cell type from the original data were not excluded. The perturb_data function was then executed to calculate the transition from the initial state (young HSCs) to the target state (old HSCs, young MPPs, or young MkPs). Only genes with significant overrepresentation were shown.

### Attention rollout across cells

From the tokenized scRNA-seq data used in Geneformer, 1000 cells were randomly selected from either young or old HSC populations. For in silico perturbed data, genes of interest were perturbed as in InSilicoPerturber of Geneformer. These were then input into the fine-tuned six-layer one-step model to compute the attention rollout (*67*). Let N represent the total number of unique genes (vocabulary size), and $n_{\text{cells}}$ represent the total number of cells analyzed. For each cell c, the gene input is represented as a sequence of length $L_{c}$, given by a vector of gene indices $g^{\left(c\right)}\in {\left\{1,\ldots ,N\right\}}^{L_{c}}$. After excluding special tokens, we obtain the effective gene indices for each cell. For each layer l∈{1,…,L} and each cell $c\in \left\{1,\ldots ,n_{\text{cells}}\right\}$, the fine-tuned model returns an attention matrix $A^{\left(c,l\right)}\in {\mathbb{R}}^{L_{c}\times L_{c}}$, where the i,j element $A_{\mathrm{ij}}^{\left(c,l\right)}$ represents the weight from token i to token j of cell c and layer l. Then, a residual self-connection is introduced

$${\tilde{A}}^{\left(c,l\right)}=\frac{1}{2}\left[A^{\left(c,l\right)}+I_{L_{c}}\right]$$ (1)

where $I_{L_{c}}\in {\mathbb{R}}^{L_{c}\times L_{c}}$ is an identity matrix.

Joint attention matrix for each cell c was computed by recursively multiplying the attention matrices across layers

$$J^{\left(c\right)}=\prod_{l=1}^{L}{\tilde{A}}^{\left(c,l\right)}$$ (2)

For each cell c, the joint attention values $J^{\left(c\right)}$ are mapped to the full vocabulary space, corresponding to all genes present in the Geneformer token dictionary. The joint attention matrix $J^{\left(c\right)}$ was then aggregated into $A_{\text{sum}}\in {\mathbb{R}}^{N\times N}$ as

$$A_{\text{sum}}\left[i,j\right]=\sum_{c=1}^{n_{\text{cells}}}J_{i_{c}j_{c}}^{\left(c\right)}$$ (3)

where $J_{\mathrm{ij}}^{\left(c\right)}$ is the attention from gene i to gene j in this cell and the sum is over all cell pairs $\left(i_{c},j_{c}\right)$, such that $g_{i_{c}}^{\left(c\right)}=i$ and $g_{j_{c}}^{\left(c\right)}=j$, wherever gene i and gene j co-occur in a cell. Last, $A_{\text{sum}}$ was normalized by the total number of cells.

### Graph visualization

To visualize transcriptional regulatory relationships extracted by attention rollout, a subgraph comprising old HSC genes, young HSC genes, and TFs associated with hematopoiesis provided in table S6 was extracted. Edges with low attention weights (thresholded at 10^−5^) were excluded, and isolated nodes were removed. Community detection was performed using the fast-greedy algorithm of the igraph package of R on an undirected version of the subgraph, with node membership and module assignment recorded as vertex attributes.

Node attributes including degree and PageRank centrality were calculated. The network was visualized using the visNetwork package.

### Quantification and statistical analysis

Data are presented as means ± SD, unless otherwise stated. For experiments involving two groups, an unpaired two-tailed Student’s *t* test was used. For multiple comparisons, one-way analysis of variance (ANOVA) followed by Tukey’s post hoc test was performed. *P* values of overlapping DEGs or DARs were calculated with a hypergeometric test using the phyper function of R program.
