# Supplementary material for: Geneformer-guided multiomics integration identifies Pbx1 as a network hub of hematopoietic stem cell aging
Source: Sci Adv. 2026 Aug 21;12(34):eaeb1346. doi: 10.1126/sciadv.aeb1346 (PMC13496190; doi:10.1126/sciadv.aeb1346)
Supplement: Supplementary file 1 [file sciadv.aeb1346_sm.pdf]

Science Advances
AAAS

Supplementary Materials for

Geneformer-guided multiomics integration identifies Pbx1 as a network hub of hematopoietic stem cell aging

Hiroshi Kobayashi *et al.*

Corresponding author: Keiyo Takubo, keiyo.takubo.e5@tohoku.ac.jp, takubo.k@jihs.go.jp, keiyot@gmail.com;
Hiroshi Kobayashi, hiroshi.kobayashi.b3@tohoku.ac.jp

Sci. Adv. **12**, eaeb1346 (2026)
DOI: 10.1126/sciadv.aeb1346

The PDF file includes:

Figs. S1 to S12
Legends for tables S1 to S10

Other Supplementary Material for this manuscript includes the following:

Tables S1 to S10

Supplementary Figures and Figure Legends

Figure S1

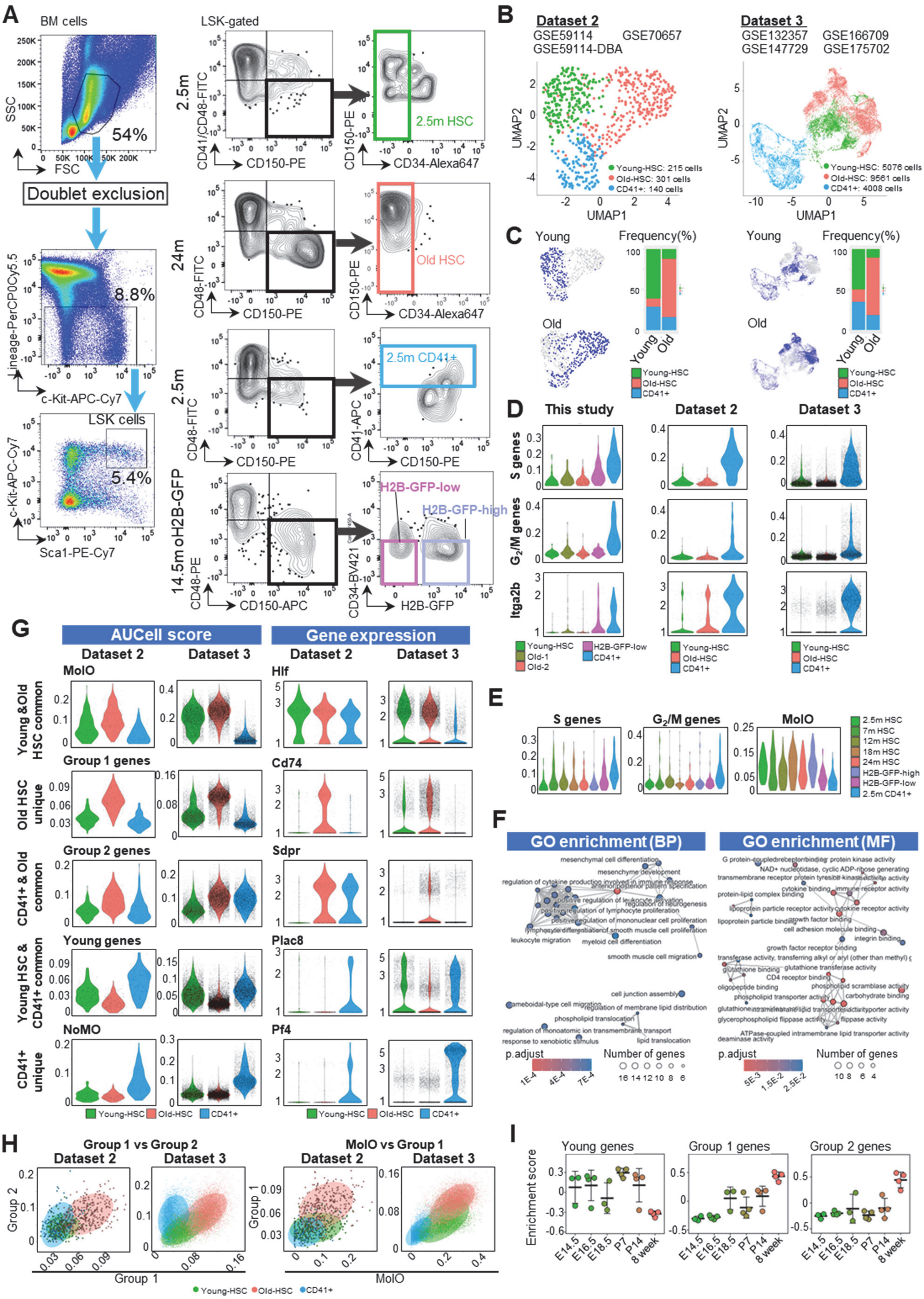

Fig. S1. Old HSCs coexist with transcriptional features of both the most primitive HSCs and megakaryocytic progenitors.

(**A**) The gating strategy for sorting young $CD150^+CD48^-CD41^-CD34^-$ LSK cells, old $CD150^+CD48^-CD34^-$ LSK cells and H2B-GFP-high or low HSCs used for scRNA-seq in Fig. 1A. (**B**) Reanalysis of existing scRNA-seq datasets, focusing on the HSC and $CD41^+$ population as indicated. Data were clustered by Seurat (**C**) Cells from either young or old mice were projected on UMAP (left). Distribution of clusters in the original group (right) are shown. Each cluster was named after the main population that composes the cluster. The cluster with high Itga2b expression was designated as CD41+. (**D**) AUCell scores of S-phase genes (top) or G_2/M-phase genes (middle) or the expression level of *Itga2b* (bottom) for indicated datasets. *P* values are provided in table S1. (**E**) AUCell scores for S-phase genes, G2/M-phase genes, and the MolO signature are shown according to the original cell source. Data are from Fig. 1. *P* values are provided in table S1. (**F**) Gene ontology (GO) analysis for Group 1 genes and clustered according to the similarity of each term. BP, biological process; MF, molecular function. (**G**) AUCell scores or expression levels in existing datasets characteristic of the five indicated groups, corresponding to Fig. 1I. (**H**) Dual-signature analysis at the single-cell level for indicated datasets. Scatter plots demonstrate the simultaneous enrichment of Group 1/MolO signatures and Group 2 signatures within individual old HSCs. Approximately 80% of cells are circled for each group. (**I**) Single-sample gene set enrichment scores for Young genes or Group 1 genes or Group 2 genes in the early developmental stage. Data are from GSE128759. *P* values are provided in table S1.

Figure S2

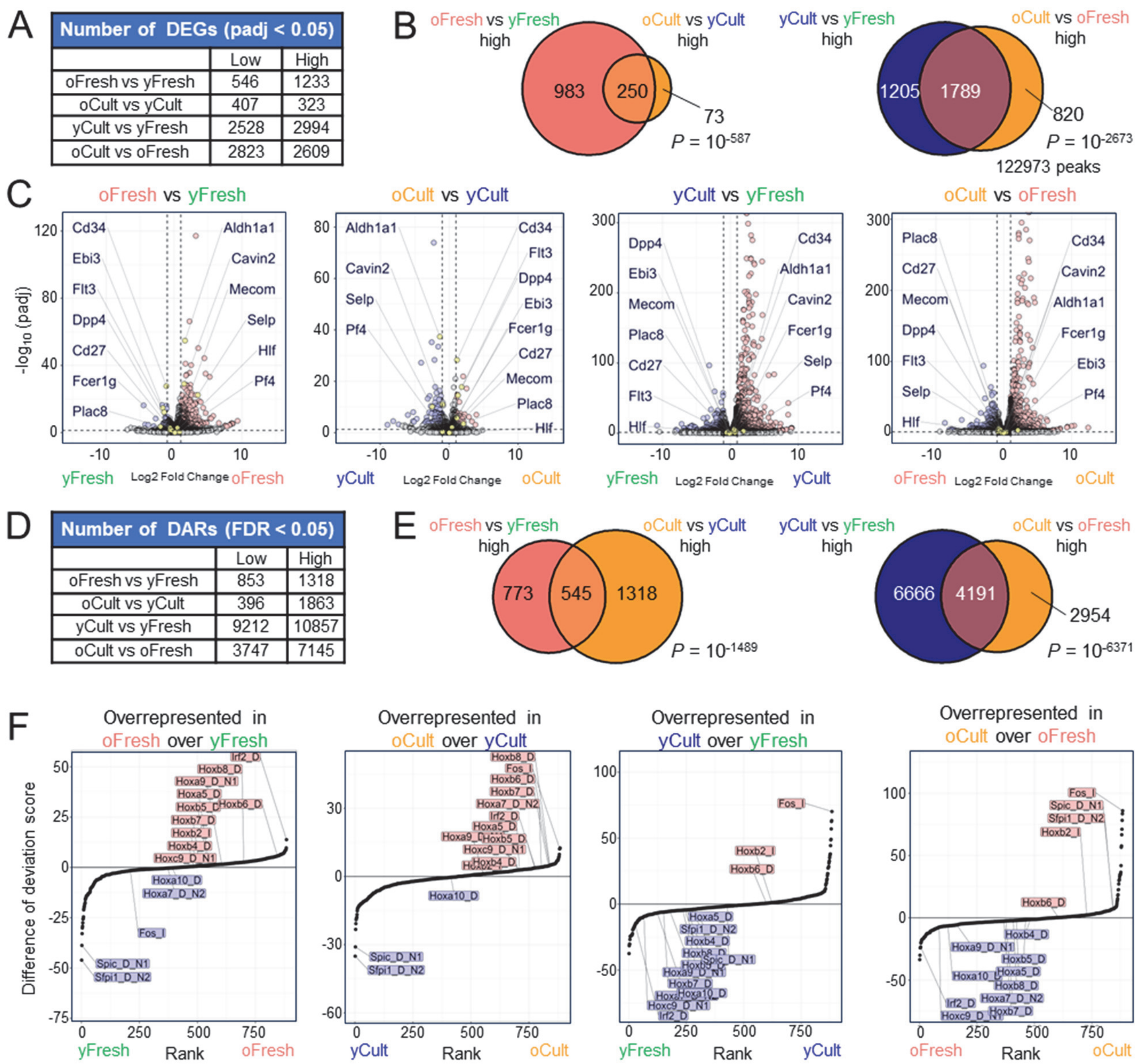

Number of DEGs (padj < 0.05)

	Low	High
oFresh vs yFresh	546	1233
oCult vs yCult	407	323
yCult vs yFresh	2528	2994
oCult vs oFresh	2823	2609

Number of DARs (FDR < 0.05)

	Low	High
oFresh vs yFresh	853	1318
oCult vs yCult	396	1863
yCult vs yFresh	9212	10857
oCult vs oFresh	3747	7145

Fig. S2. Old HSCs are defective in activating differentiation programs after cytokine stimulation. (**A**) The number of differentially expressed genes (DEGs) with $p_{adj} < 0.05$, comparing indicated groups of data, presented in Fig. 2. (**B**) Overlap of genes highly expressed in old samples, either in fresh or cultured conditions, compared with young HSCs (top), or genes that are highly expressed in the cultured group, in either young or old HSCs, compared with fresh samples (bottom). (**C**) Volcano plots of DEGs. (**D**) The number of differentially accessible regions (DARs) with FDR < 0.05 comparing indicated groups of data presented in Fig. 2. (**E**) Overlap of DARs, with peaks that are elevated in old samples under either fresh or cultured conditions compared to young HSCs (top) or DAR s that exhibit higher signal in cultured groups for both young and old samples compared to fresh samples (bottom). (**F**) Fold enrichment of TF-binding motifs between indicated groups estimated by chromVAR analysis using the mouse_pwms dataset.

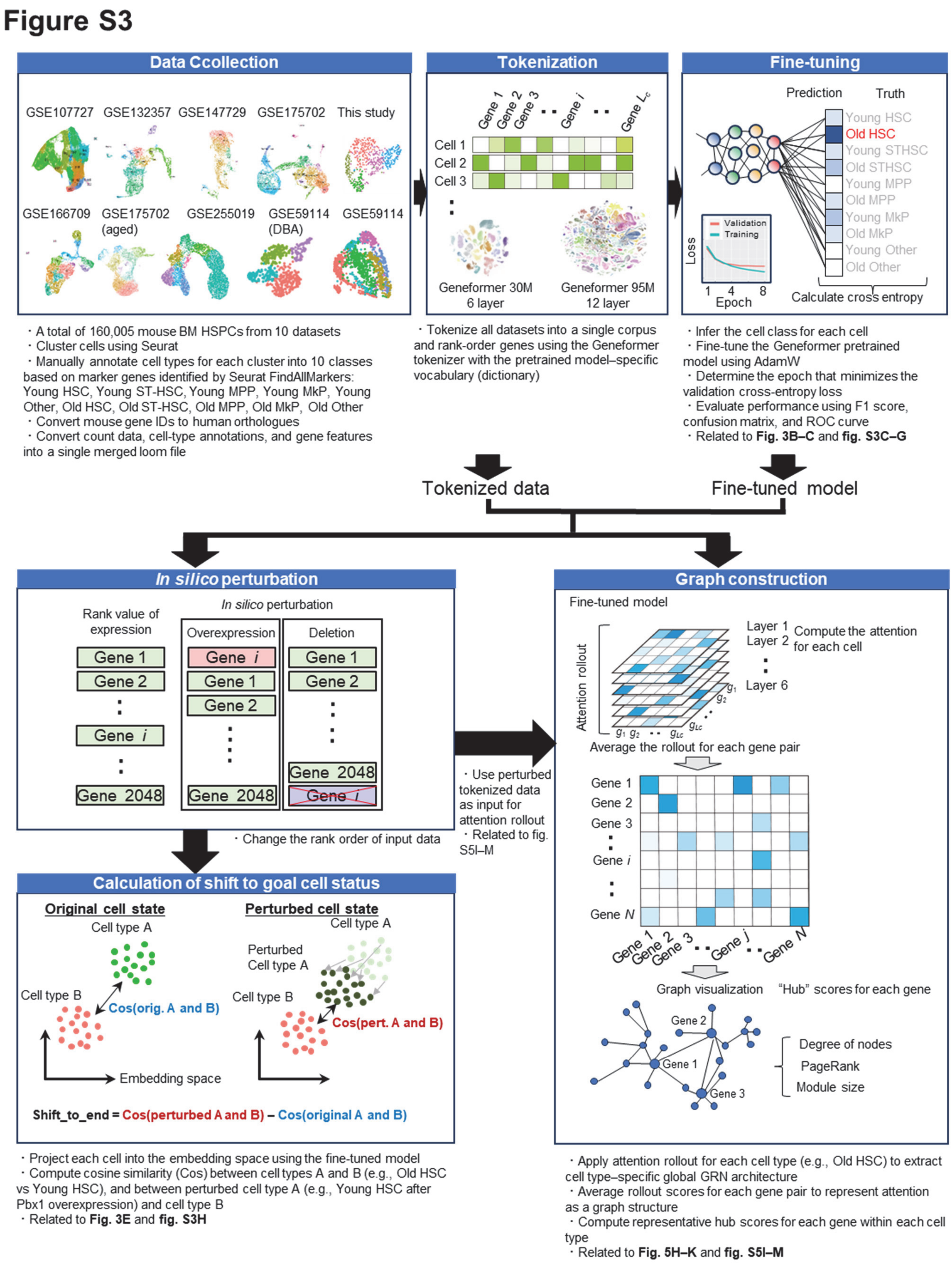

Fig. S3. An overview on analysis pipeline of Geneformer described in this study.

Overview of the Geneformer analysis pipeline used in this study. Collected datasets were converted into single-tokenized data and fine-tuned to predict predefined "true" manually annotated classifications. Subsequently, either *in silico* perturbations were performed to identify genes with

strong influence on alterations in differentiation/aging trajectories, or higher-order regulatory architecture was extracted and represented as a graph to visualize the gene regulatory network (GRN).

Figure S4

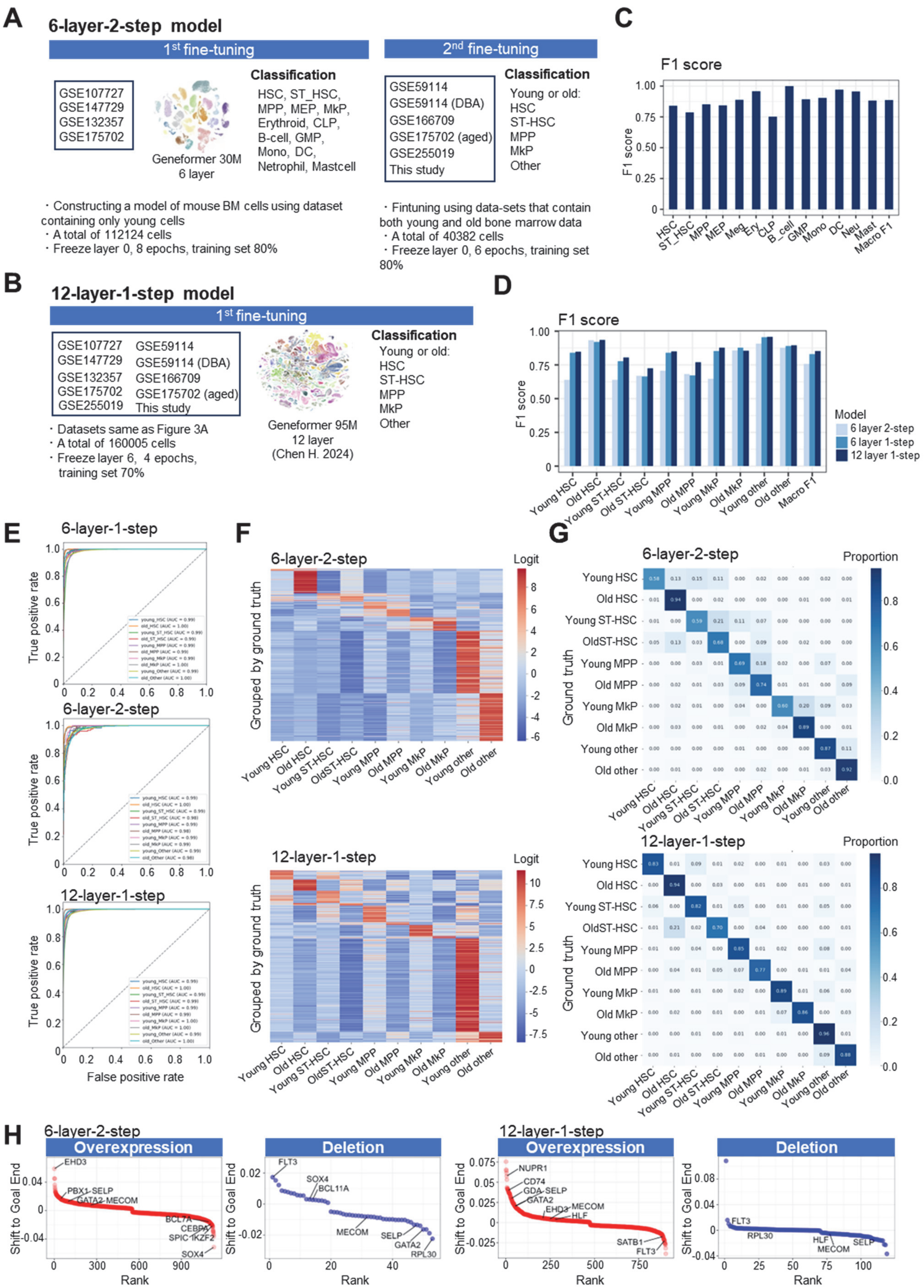
A
6-layer-2-step model
1st fine-tuning
GSE107727
GSE147729
GSE132357
GSE175702
Geneformer 30M
6 layer
Classification
HSC, ST_HSC, MPP, MEP, MkP, Erythroid, CLP, B-cell, GMP, Mono, DC, Netrophil, Mastcell
・Constructing a model of mouse BM cells using dataset containing only young cells
・A total of 112124 cells
・Freeze layer 0, 8 epochs, training set 80%
2nd fine-tuning
GSE59114
GSE59114 (DBA)
GSE166709
GSE175702 (aged)
GSE255019
This study
Classification
Young or old:
HSC
ST-HSC
MPP
MkP
Other
・Fintuning using data-sets that contain both young and old bone marrow data
・A total of 40382 cells
・Freeze layer 0, 6 epochs, training set 80%
B
12-layer-1-step model
1st fine-tuning
GSE107727 GSE59114
GSE147729 GSE59114 (DBA)
GSE132357 GSE166709
GSE175702 GSE175702 (aged)
GSE255019 This study
Geneformer 95M
12 layer
(Chen H. 2024)
Classification
Young or old:
HSC
ST-HSC
MPP
MkP
Other
・Datasets same as Figure 3A
・A total of 160005 cells
・Freeze layer 6, 4 epochs, training set 70%
C
F1 score
F1 score
HSC ST_HSC MPP MEP Meg Ery CLP B_cell GMP Mono DC Neu Mast Macro F1
D
F1 score
F1 score
Model
6 layer 2-step
6 layer 1-step
12 layer 1-step
Young HSC Old HSC Young ST-HSC Old ST-HSC Young MPP Old MPP Young MkP Old MkP Young other Old other Macro F1
E
6-layer-1-step
6-layer-2-step
12-layer-1-step
True positive rate
False positive rate
F
6-layer-2-step
12-layer-1-step
Logit
Grouped by ground truth
Young HSC Old HSC Young ST-HSC OldST-HSC Young MPP Old MPP Young MkP Old MkP Young other Old other
G
6-layer-2-step
12-layer-1-step
Proportion
Ground truth
Young HSC Old HSC Young ST-HSC OldST-HSC Young MPP Old MPP Young MkP Old MkP Young other Old other
H
6-layer-2-step
12-layer-1-step
Overexpression
Deletion
Shift to Goal End
Rank
EHD3
PBX1 SELP
GATA2 MECOM
BCL7A
CEBPA
SPIC IKZF2
SOX4
FLT3
SOX4
BCL11A
MECOM
SELP
GATA2
RPL30
NUPR1
CD74
GDA SELP
GATA2
EHD3 MECOM
HLF
SATB1
FLT3
FLT3
RPL30
HLF
SELP
MECOM

Fig. S4. Fine-tuning of a pretrained Geneformer enables identification of key transcriptional networks in old HSCs.

(**A**) An overview of 2-step model training using 6-layer 30 M Geneformer. (**B**) An overview of 1-step model training using 12-layer 95M Geneformer. (**C**) F1 scores for each class as well as macro F1 after fine-tuning with young BM HSPC datasets in fig. S4A. (**D**) Comparison of F1 scores for each class across the indicated three models. (**E**) Receiver Operating Characteristic (ROC) curves and Area Under the Curve (AUC) scores for the indicated models. (**F**) Heatmap representation of logit scores for class predictions by the indicated models grouped, organized according to the ground truth. (**G**) Confusion matrix for classification of the indicated models. (**H**) Estimated transcriptional deviation from young HSCs to old HSCs after *in silico* perturbation using either the 6-layer-2-step model or the 12-layer-1-step model. Shown are significant genes.

Figure S5

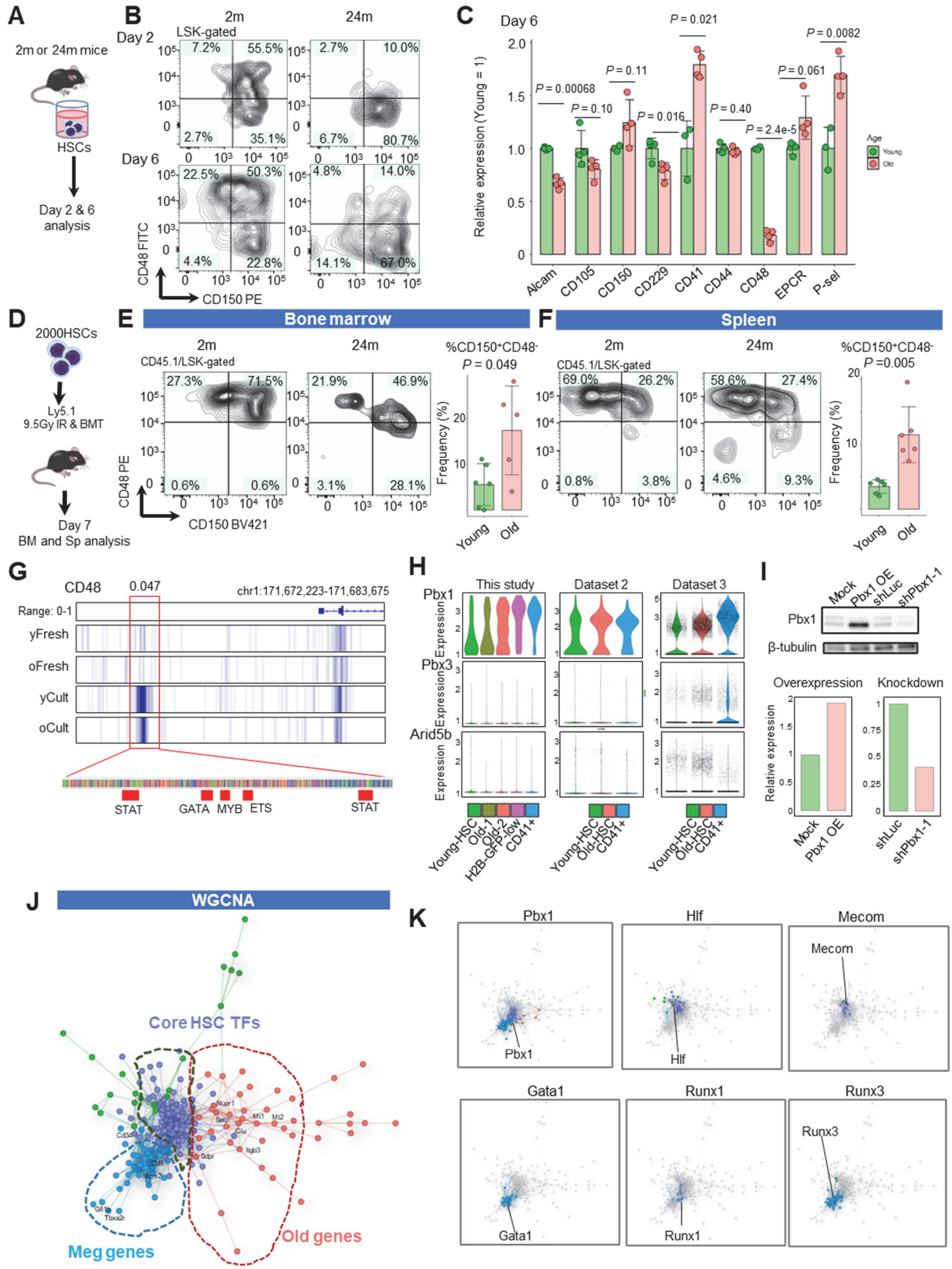

Fig. S5. Identification of Pbx1 as a key TF that confers age-related transcriptional features upon HSCs.

(**A**) Experimental design for HSC culture. (**B**) Representative FACS plots of the LSK fraction from 2-month (left) or 24-month mice cultured after 2 days (top) or 6 days (bottom). The frequency of subpopulation based on CD150 and CD48 expression is shown. (**C**) Relative mean fluorescence intensity of surface markers in young or old HSCs cultured for 6 days (mean ± SD, n = 4). Unpaired two-tailed t-test. (**D**) Experimental design for transplantation of HSCs. (**E**) Representative FACS plots and the frequency of the HSC fraction in the donor-derived LSK fraction in recipient bone marrow (mean ± SD, n = 5 or 6). Unpaired two-tailed t-test. (**F**) Representative FACS plots and the frequency of the HSC fraction in the donor-derived LSK fraction in recipient spleen (mean ± SD, n = 5 or 6). Unpaired two-tailed t-test. (**G**) Heatmap of ATAC signal in a genomic region upstream of the CD48 gene. An approximately 300-bp region of DARs overrepresented in yCult group is magnified. Putative TF binding sites are shown. Number above the box represents *P* values estimated by edgeR comparing yCult and oCult. (**H**) Expression levels of Pbx1, Pbx3, and Arid5b in indicated scRNA-seq datasets. *P* values are provided in table S1. (**I**) Western blot for Pbx1 or β-tubulin (internal standard) in $CD150^+$ LSK cells transduced with indicated vectors. Bars (bottom) indicate quantification of the fold change of Pbx1/β-tubulin ratio. (**J**) Young HSC genes, old HSC genes (both are from Fig. 1G), and TFs used in Fig. 4A were combined and WGCNA was performed using scRNA-seq data shown in Fig. 1. Each cluster was named after its representative genes. (**K**) Network adjacent to representative genes associated with HSC or megakaryocytic program.

Figure S6

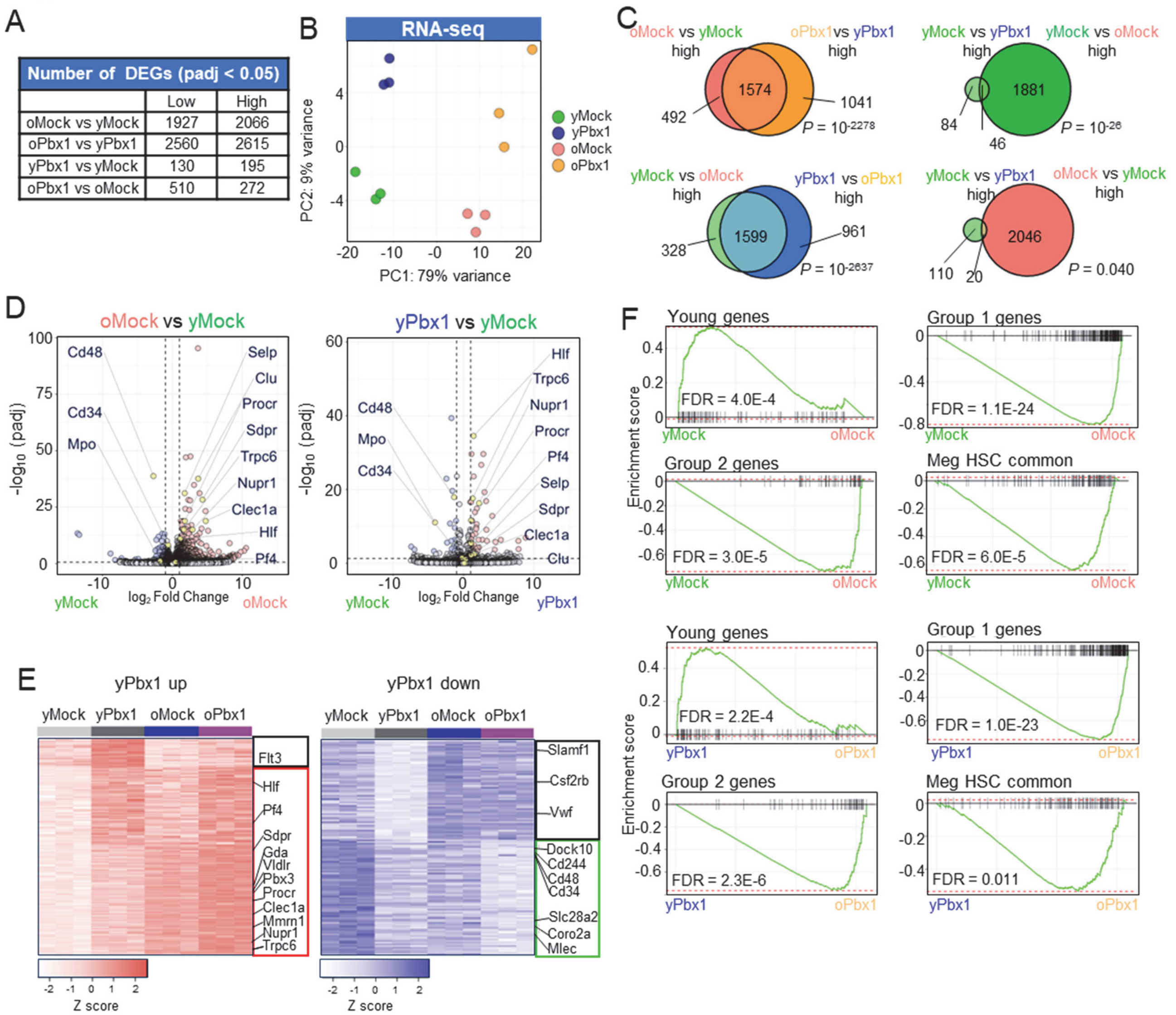

Fig. S6. Pbx1 activates a core transcriptional network shared by aging and stemness

(**A**) The number of differentially expressed genes (DEGs) with $p_{adj} < 0.05$ comparing indicated groups of data presented in Fig. 5A. (**B**) PCA of RNA-seq using normalized read counts. (**C**) Overlap of genes highly expressed in the indicated comparisons. *P* values were assessed by a hypergeometric test. (**D**) Volcano plots of DEGs comparing indicated groups. (**E**) Heatmap representation of z-normalized gene expression in RNA-seq data following k-means clustering of DEGs with elevated expression (left) or downregulated (right) in the yPbx1 group relative to the yMock group. DEGs exhibiting elevated expression in oMock compared to yMock are highlighted with red boxes and DEGs exhibiting elevated expression in yMock compared to oMock are highlighted with blue boxes. (**F**) Gene set enrichment analysis comparing yMock and oMock (top) or yPbx1 and oPbx1 (bottom) for indicated gene sets.

Figure S7

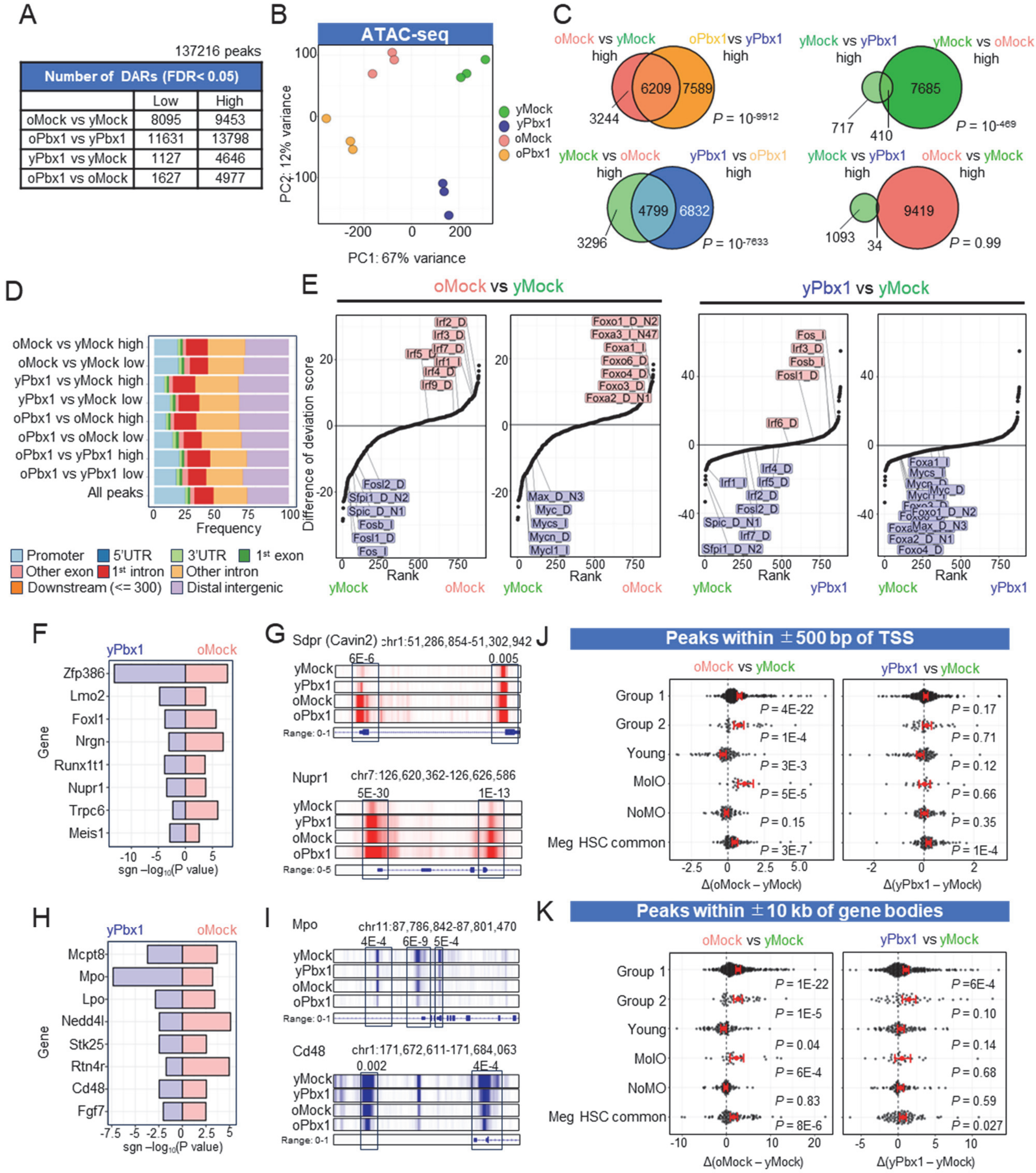

Fig. S7. Pbx1 open chromatin regions of Group1 genes Megakaryocyte-HSC common genes. (**A**) The number of DARs with FDR < 0.05 comparing indicated groups of data presented in Fig. 5A. (**B**) PCA of ATAC-seq using normalized read counts. (**C**) The Intersection of DARs for indicated comparisons. (**D**) The percentage of annotated genomic regions on which each DAR locates. (**E**) Differences in deviation of motifs between the oMock and yMock groups (left) or yPbx1 and yMock groups (right), estimated by chromVAR analysis using the mouse_pwms dataset. yFresh-specific Spi1 motifs, oFresh-specific Irf and AP-1, FoxO motifs, and Myc motifs are highlighted. (**F**) Signed log p-

values of GREAT-predicted genes associated with DARs highly accessible in the yPbx1 group compared with the yMock group are shown with results comparing oMock and yMock. (**G**) Representative genomic loci highly accessible in both yPbx1 and oMock. *P* value between yMock and yPbx1 is shown above the boxed region. (**H**) Signed log p-values of GREAT-predicted genes associated with DARs poorly accessible in the yPbx1 group compared with the yMock group are shown with results comparing yMock and oMock. (**I**) Representative genomic loci poorly accessible in both yPbx1 and oMock. The *P* values between yMock and yPbx1 is shown above the boxed region. (**J**) Differences in the summed ATAC-seq signal around promoters (TSS ± 500 bp) for genes in the indicated gene sets, comparing oMock versus yMock (left) or yPbx1 versus yMock (right). Two-sided Wilcoxon rank-sum test. (**K**) Differences in the summed ATAC-seq signal around promoters (peaks within ±10 kb of the nearest genes, including gene bodies) for genes in the indicated gene sets, comparing oMock versus yMock (left) or yPbx1 versus yMock (right). Red dots and error bars indicate the mean ± standard error, with standard errors estimated by bootstrap resampling using the mean_cl_boot function. Statistical significance was evaluated using a two-sided Wilcoxon rank-sum test.

Figure S8

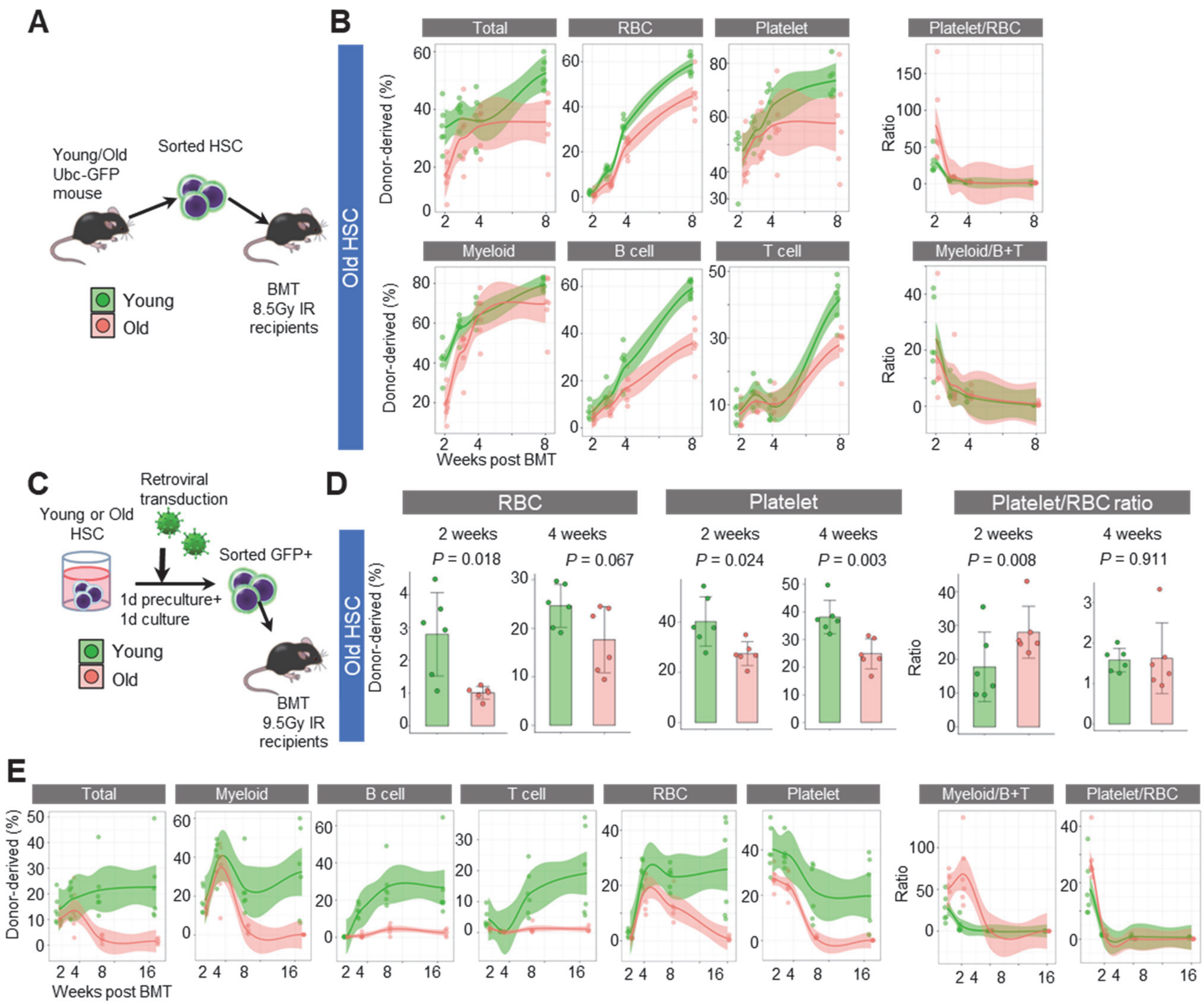

Fig. S8. Old HSCs display reduced red blood cell output and an early platelet bias after BMT. (**A**) Experimental design. Continuation of the data shown in Fig. 6, A and B. (**B**) Frequency or ratio of the indicated fractions in peripheral blood (PB) after BMT (n = 6 per group from a single experiment, lowess spline with shaded 95% CI). *P* values are provided in table S1. (**C**) Retroviral expression of GFP with pMYs-IRES-GFP vector in young or old HSCs. Each of 300 sorted GFP^{+} cells 1 day after transduction were transplanted to irradiated mice. (**D**) Frequency of donor-derived RBCs and platelets, and the platelet/RBC ratio in PB after BMT (mean ± SD, n = 6 from a single experiment), unpaired two-tailed t-test. (**E**) The frequency or ratio of indicated fractions in peripheral blood (PB) after BMT (n = 6 for each group from a single experiment. Lowess spline, shadow indicates 95% CI). *P* values are provided in table S1.

Figure S9

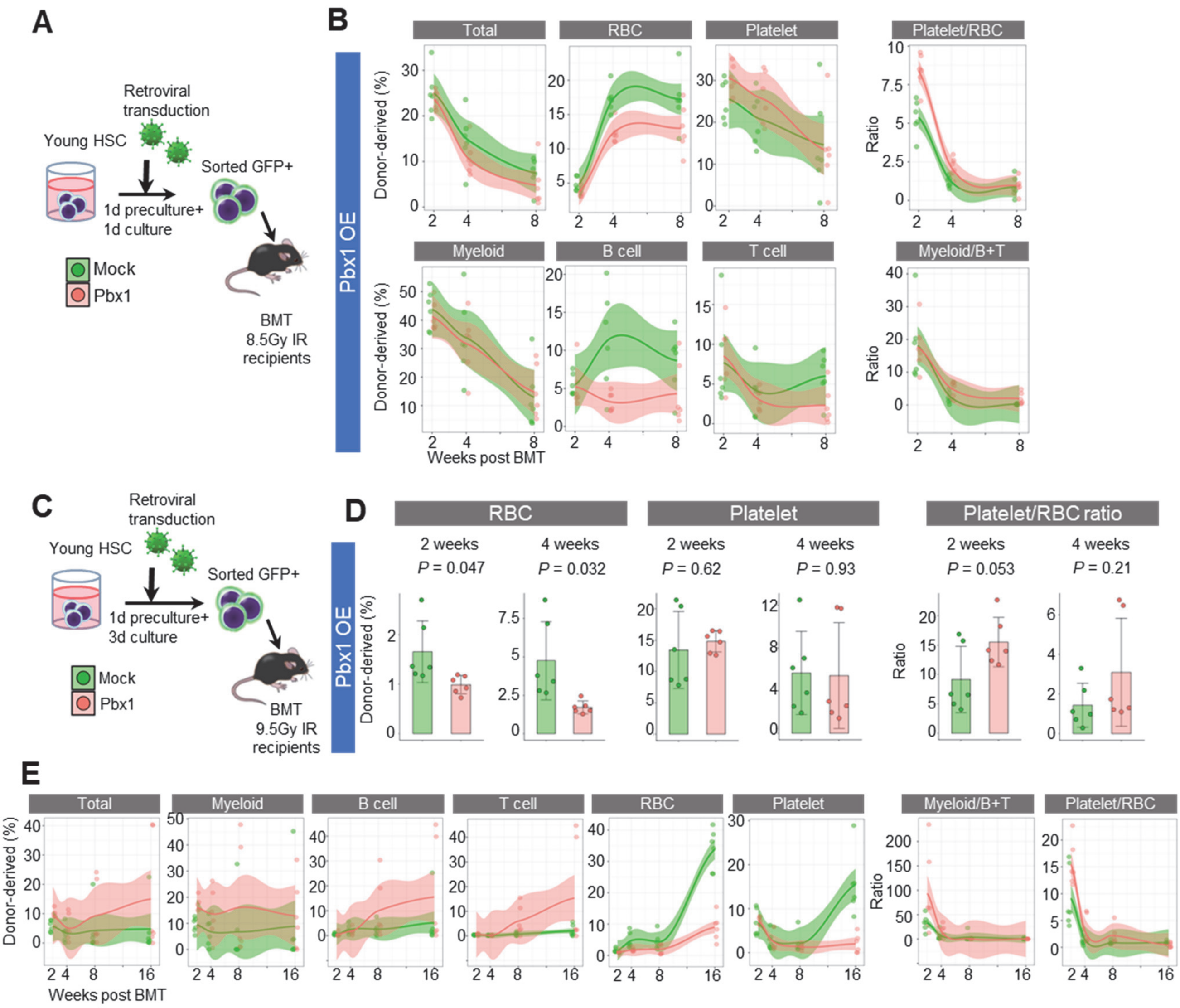

Fig. S9. Pbx1 is responsible for platelet bias in old HSCs

(**A**) Experimental design. Continuation of the data shown in Fig. 6, C and D. (**B**) Frequency or ratio of the indicated fractions in peripheral blood (PB) after BMT (n = 6 per group from a single experiment, lowss spline with shaded 95% CI). *P* values are provided in table S1. (**C**) Mock or Pbx1 was retrovirally transduced in young HSCs and 400 GFP^{+} cells 3 days after transduction were transplanted to irradiated mice. (**D**) Frequency of donor-derived RBCs and platelets, and the platelet/RBC ratio in PB after BMT (mean ± SD, n = 6 from a single experiment), unpaired two-tailed t-test. (**E**) The frequency or ratio of indicated fractions in peripheral blood (PB) after BMT (n = 6 for each group from a single experiment. Lowess spline, shadow indicates 95% CI). *P* values are provided in table S1.

Figure S10

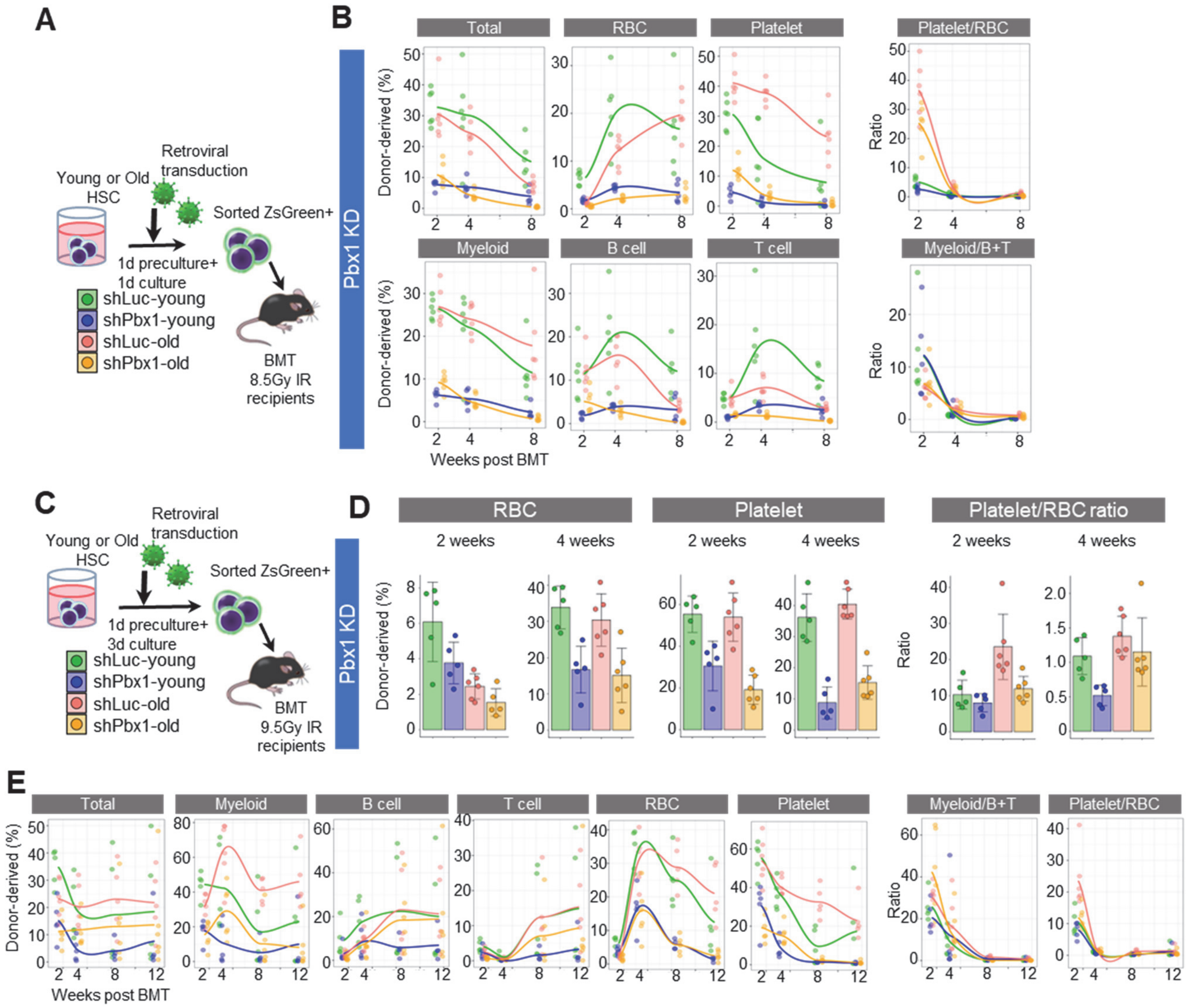

Fig. S10. Pbx1 is responsible for platelet bias in old HSCs

(**A**) Experimental design. Continuation of the data shown in Fig. 6, E and F. (**B**) Frequency or ratio of the indicated fractions in peripheral blood (PB) after BMT (n = 6 per group from a single experiment, lowess spline with shaded 95% CI). *P* values are provided in table S1. (**C**) Either shLuc or shPbx1 was retrovirally transduced in young or old HSCs and 400 Zs-Green^{+} cells 3 days after transduction were transplanted into irradiated mice. (**D**) Frequency of donor-derived RBCs and platelets, and the platelet/RBC ratio in PB after BMT (mean±SD, n = 6 from a single experiment). *P* values are provided in table S1. (**E**) The frequency or ratio of indicated fractions in peripheral blood (PB) after BMT (n = 6 for each group from a single experiment. Lowess spline). *P* values are provided in table S1.

Figure S11

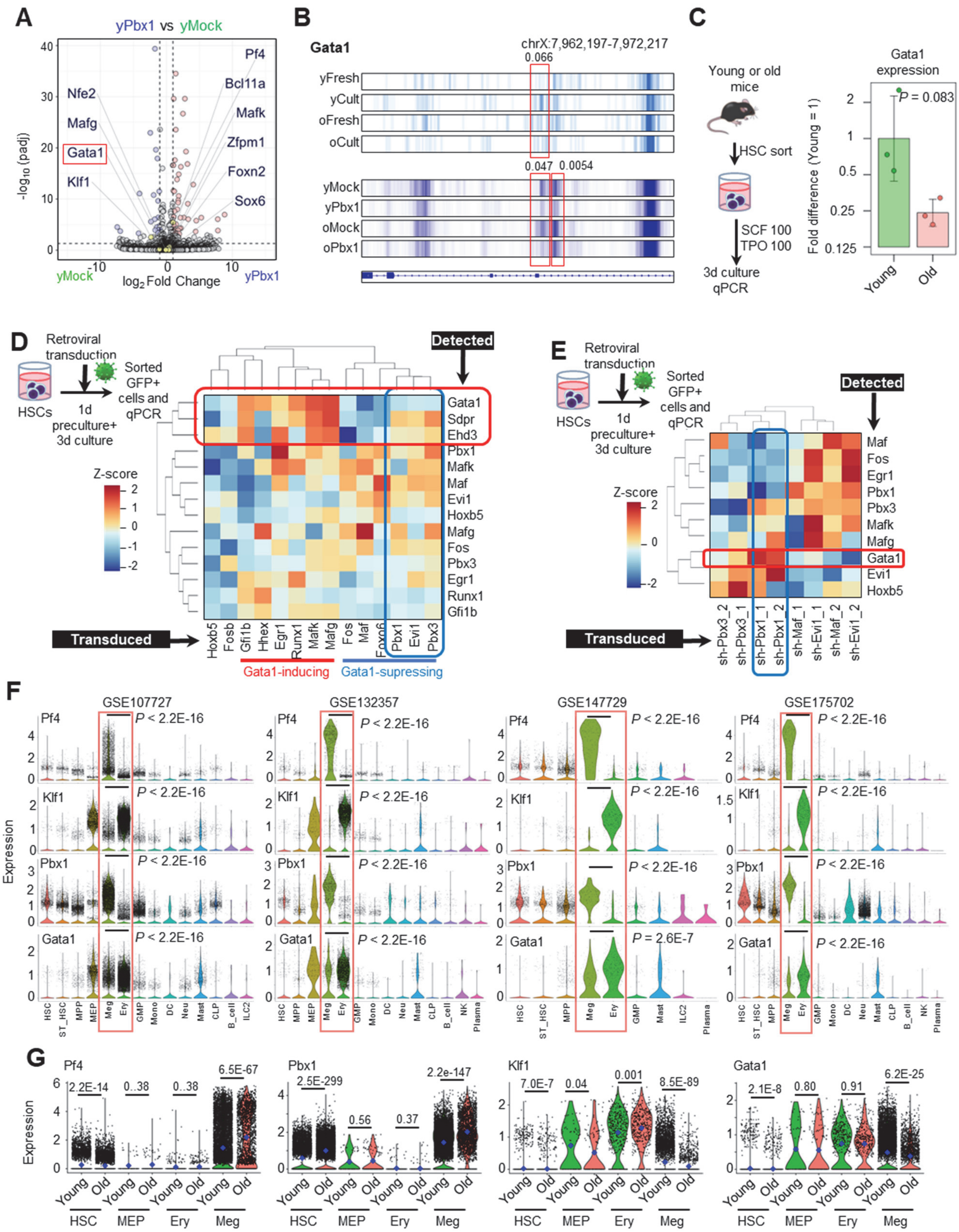

Fig. S11. Pbx1 suppresses Gata1 expression.

(**A**) The RNA-seq data of Fig. 5A comparing yMock and yPbx1, and either megakaryocyte or erythroid genes are highlighted. (**B**) Normalized ATAC-seq signals of Fig. 2 (top) and Fig. 5 (bottom) around the

Gata1 gene. Numbers above the box represent *P* values estimated by edgeR comparing yFresh and yCult (top) and yMock and yPbx1(bottom). (**C**) HSCs from either young (2-month) or old (18-month) mice were sorted and cultured for 3 days in SF-O3, and Gata1 expression was examined by qPCR (mean ± SD, n = 3 biological replicates), unpaired two-tailed t-test. (**D**) qPCR after young HSCs were retrovirally transduced with indicated genes. Z-scores of expression relative to Mock transduced samples, normalized to Gapdh expression are shown. The red square indicates Gata1 and old HSC genes (Sdpr and Ehd3). Expression levels of genes that cannot be distinguished from transduced factors are set to 0. The blue square indicates genes suppressing Gata1 while maintaining old HSC genes (each tile represents a single sample; 3 experiments were combined). (**E**) qPCR after young HSCs were retrovirally knocked down by shRNA vector for indicated genes. Z-score are shown. The red square indicates Gata1, and the blue square indicates Pbx1 knockdown vector. (Each tile represents a single sample; 2 experiments were combined) (**F**) Gene expression levels of Pf4, Klf1, Pbx1, and Gata1 in each population of scRNA-seq datasets. (**G**) scRNA-seq datasets from GSE166709, GSE175702, GSE255019, GSE107727, GSE132357, and GSE147729 were integrated. Cells annotated as HSCs, MEPs, erythroid (Ery) cells, and megakaryocytic lineage (Meg) cells from young or old mice were extracted, and the expression levels of the indicated genes were evaluated. Blue dots denote the mean expression level. Statistical comparisons were conducted using an unpaired two-tailed Student's t-test.

Figure S12

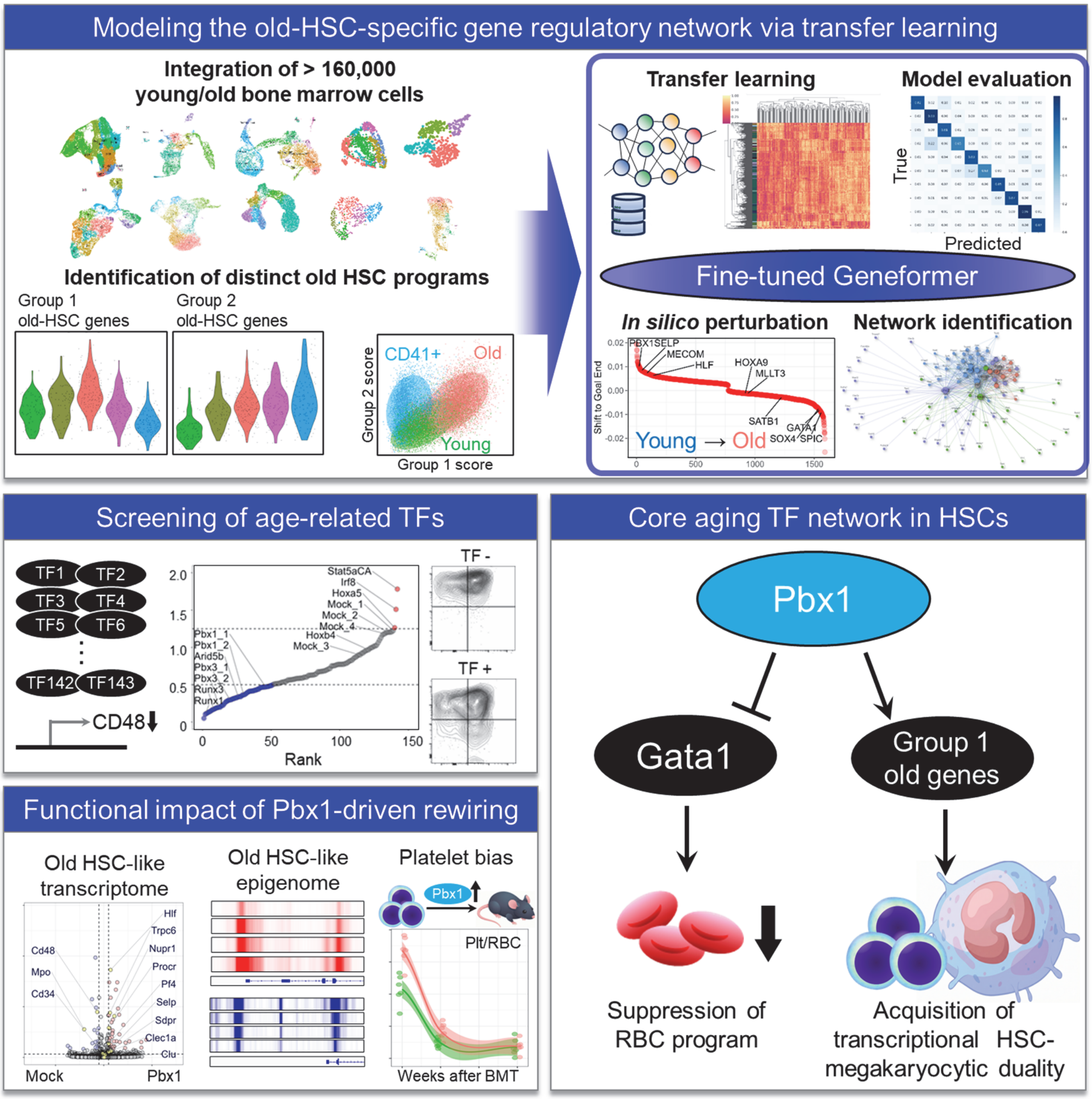

Fig. S12. Illustration of experimental design and conclusion.

The summary of the overall design and the conclusion from the study is illustrated.

Supplementary Tables Legends

Table S1 Summary of *P* values for the data presented in the figures.

P values are reported. For two-group comparisons, unpaired two-tailed t-tests were performed. For multiple comparisons, Tukey's post hoc test following one-way ANOVA was used.

Table S2 Custom gene sets used for gene set enrichment analysis.

Genes associated with HSPCs under various conditions are listed in the GMT format.

Table S3. Alignment metrics of ATAC-seq samples.

Alignment metrics including total reads, aligned reads, and percent of duplicates of ATAC-seq samples using bowtie2 are listed.

Table S4. Genes used for retroviral overexpression in the TF screening.

Genes associated with HSPC and differentiated cell functions are subcloned into retroviral vectors. Genes and their accession are listed.

Table S5. Efficiency of overexpression and knockdown.

Fold changes in expression levels following retroviral overexpression or knockdown, as quantified by qPCR, are summarized.

Table S6. Genes used for network analysis.

Young HSC gene, old HSC gene, and TFs used for screening which were subjected to network analysis are listed.

Table S7. Transduction efficiency of transplanted HSCs prior to sorting.

The frequencies of successfully transduced GFP- or ZsGreen-positive HSCs prior to sorting and transplantation are summarized.

Table S8. Oligonucleotide sequences of qPCR, genotyping, and shRNA.

qPCR primers, shRNA targets, and primers for genotyping pTRE-H2BGFP as well as Tal1-tTA mice are listed.

Table S9. Parameters used in scRNA-seq analysis.

Parameters used for scRNA-seq analysis using the Seurat package is listed.

Table S10. Parameters used for training Geneformer.

Parameters used for models of Geneformer used are listed.
